# Comparison of Environmental Impact and Nutritional Quality among a European Sample Population – findings from the Food4Me study

**DOI:** 10.1038/s41598-018-20391-4

**Published:** 2018-02-05

**Authors:** Christie Walker, Eileen R. Gibney, Stefanie Hellweg

**Affiliations:** 1 Institute of Environmental Engineering, ETH Zurich, 8093 Zürich, Switzerland; 2 Institute of Food and Health, University College Dublin, Dublin 4, Ireland

## Abstract

This study evaluates the relationship between environmental impacts and diet quality through several environmental and nutritional indicators, using data from over 1400 participants across seven European countries in the Food4Me study. Comparisons of environmental impacts and dietary quality were evaluated across country, gender groups, and dietary patterns. While there was clear variability within the different subsets, there were large differences observed in both dietary quality and environmental impacts between cultures, genders, and dietary patterns. Individuals abstaining from red meat consistently had lower impacts in combination with lower consumption of harmful nutrients (saturated fats, sodium, and sugars) while maintaining average intake of beneficial nutrients. A ‘best practice’ diet with low impacts, adequate nutrient intake, and low saturated fats, sodium, and sugars, was constructed from the sample and used as a benchmark. Recorded eating patterns were compared to this recommended diet. On average, intakes of sweets, meats, and drinks should be decreased and intakes of vegetables and cereals increased, at varying rates depending on country and gender. However, the study shows a large spread of eating patterns and recommendations for lowering environmental impacts and increasing nutritional quality vary greatly among individuals.

## Introduction

It has been well documented that food consumption significantly contributes to an individual’s environmental impact^1^, however the relationship between a food’s impact and its function is less established^2^. To date, impact reductions have focused on food production processes, however eating patterns, the drivers behind production demand, need to also be understood^3^. For example, the growing trend of Western eating patterns, typically characterized by high meat intakes, are associated with high climate change impacts^4,5^. As there is no healthy food per se, but rather healthy diets and eating patterns^6^, there is importance in considering full, practiced diets. This paper focuses on the role of food choices due to culture^7,8^, gender^9^, and three dietary pattern choices (no meat and fish diets, no red meat diets, and no dairy diets) in shaping an individual’s environmental impact and dietary quality.

Previous research has been conducted examining environmental impacts associated with hypothetical dietary patterns (e.g. vegetarian) or hypothetical diets meeting given recommendations^5,10,11^, food availability (based on a country’s import and export ratios and/or purchasing data)^12,13^, and impacts at a meal level^14–16^, however data examining impacts from an individual’s habitual diet in combination with nutritional effects is limited^17–19^ and often based only on one specific country. Evaluating habitual diets allows for a direct comparison between an individual’s environmental impact and nutritional quality, as studies involving only hypothetical dietary patterns may miss the nuances associated with self-selected food intake, and studies utilizing food availability cannot measure the nutrient intake at an individual level.

Food related environmental assessments often use climate change as the sole environmental impact indicator^2^, however the evaluation of other indicators when analyzing food production is necessary. For example, crop growth (for both direct human consumption and as livestock feed) causes global stress on both water and land resources, the effects of which are water scarcity and land degradation^20^. Biodiversity loss, attributed to both freshwater consumption and land use, is largely associated with agriculture activities^21^. Habitat loss through land use is the primary driver of biodiversity loss^22^. The rate of biodiversity loss, an impact largely unquantified in past and current food studies, is one of the nine planetary boundaries, and is currently being exceeded at a far higher rate than the other eight boundaries (including climate change), which warrants its inclusion in food impact assessments^23^. This research will quantify not only the climate change impacts from an individual’s food intake, but also the water scarcity footprint and land-use driven biodiversity loss.

Several indicators have been proposed to measure the quality of a person’s diet^24–26^. These indicators aim to give an overall description of an individual or population diet with respect to recommended intakes and often correlate the risk of various diseases to understand the link between diet and disease^27^. For example, in developed regions such as Western Europe, the Global Burden for Disease has found that a combination of under-consumption of primarily whole grains, omega 3 fatty acids, and fruits in combination with excessive intake of red and processed meats and sodium contribute to the region’s Disability Adjusted Life Years (DALYs)^28^. Taken together, dietary risks contributed to over 10% of Western Europe’s DALYs^28^, and recently methods of combining human health impacts (measured as DALYs) due to food production and dietary related diseases using the Global Burden of Disease results have been proposed^29^.

This paper aims to fill several research gaps. Firstly, environmental impacts will be investigated using reported dietary intakes from Food4Me, which represent real rather than hypothetical diets^30^. These estimates of individual dietary intakes are from a study of adults who were broadly representative of the seven European countries from which they were recruited^31^, and allow for assessment of impact and diet quality variability both within and among groups, and an evaluation of impacts in countries that have not yet been largely investigated. Secondly, often overlooked impact categories such as water scarcity footprint and biodiversity loss will be included in the assessment, and drivers of these impacts investigated. Thirdly, a sensitivity of an individual’s diet to impacts associated with food waste, a growing topic and clear way to reduce impacts^32^, will be analyzed. Finally, the relationship between diet quality and impacts will be explored, and the potential impact reduction of dietary changes assessed.

## Results and Discussion

### Nexus of Environmental Impacts and Diet Quality

This study included over 1400 European adults of both genders and a range of ages who monitored their food intake over the course of a month (for details see Methods section and Livingstone *et al*.^31^). Environmental impacts and nutrient consumption were calculated from the study participant’s recorded food intake.

**Figure 1:**
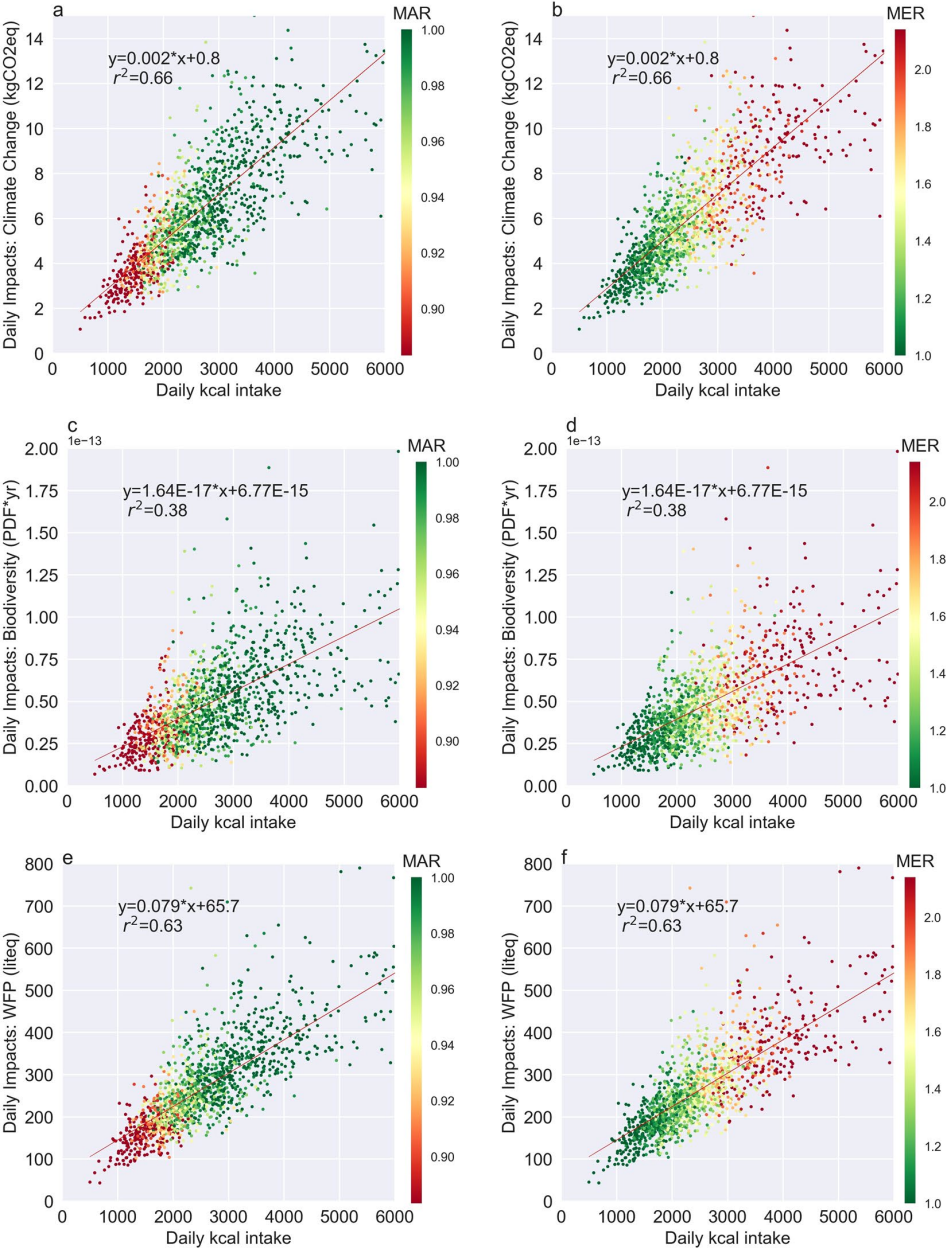
Daily energy intake compared to each of the investigated impact categories. (**a** and **b)** show climate change impacts, (**c** and **d**) show biodiversity loss, and e and f show water scarcity footprint. Shading on the graphs in the left column (**a**,**c** and **e**) represent the mean adequacy ratio (MAR) of beneficial nutrients and shading on the graphs in the right column (**b**,**d** and **f**) represent the mean excess ratio (MER) of saturated fats, sugars, and sodium. Each point represents one individual. The equation represents the linear correlation between energy intake and impact, and the r^2^ value represents the coefficient of determination.

Three environmental impact categories (climate change, water scarcity footprint, and biodiversity loss) were investigated within this paper. In general, foods/dishes with a high impact in one category tended to have high impacts in the other categories, meaning that impacts among the different impact categories were fairly well correlated (kgCO_2_eq impacts compared to biodiversity loss and water scarcity footprint had r^2^ values of 0.79 and 0.72, respectively). Figure 1 shows the relationship of each of the impact categories (Fig. 1a,b for climate change, Fig. 1c,d for biodiversity loss, and Fig. 1e,f for water scarcity footprint) to the sample population’s daily energy intake. Energy intake was correlated to all three environmental impacts, but r^2^ values varied, with the best correlation with energy intake being found for climate change (Fig. 1a,b), and the worst for biodiversity (Fig. 1c,d). The vertical spread in data shows the differences in each impact due to varying eating patterns, and it demonstrates that, in spite of the correlation to energy intake, for the same calorie intake the impacts can differ by factors of 4, 9, and 4, for impacts of climate change, land-use related biodiversity loss and water scarcity, respectively. Differences in severity of impacts among the impact categories for an individual could be attributed to an individual’s higher intake of food items originating from tropical countries (such as chocolate or coffee), as items from such tropical regions tend to have relatively higher biodiversity impacts when compared to the climate change impacts or water scarcity footprint. Other differences could be attributed to the impacts associated with energy use in processing, which are considered only in the climate change impact category (Supplementary Electronic Table).

Nutritional quality (measured as the mean adequacy ratio (MAR) of 19 beneficial nutrients and mean excess ratio (MER) of 3 harmful nutrients [saturated fats, sugars, and sodium]) was highly correlated with kcal intake, as expected^33,34^. There is a rather small range of kcal intake, between 2100 and 2300 kcal, as can be seen in Fig. 1, at which the mean adequacy ratio is maximal, the mean excess ratio is minimal, and lower to average impacts are achieved. However, each individual’s energy requirements will vary from this based on physiological state^35^, gender^36^, and physical activity^37^ levels. Below this energy intake, the mean adequacy ratio tended to sharply decline with decreasing kcal intake and above it the mean excess ratio began to increase. This is not meant to indicate an all-encompassing energy intake suggestion, but rather simply the observed boundary, based on self-reported energy intakes, for low and high environmental impacts in combination with nutrient intake. Above 2300 kcal, 69% of males and 47% of females had impacts exceeding the upper third of impacts in their respective subsets.

**Figure 2:**
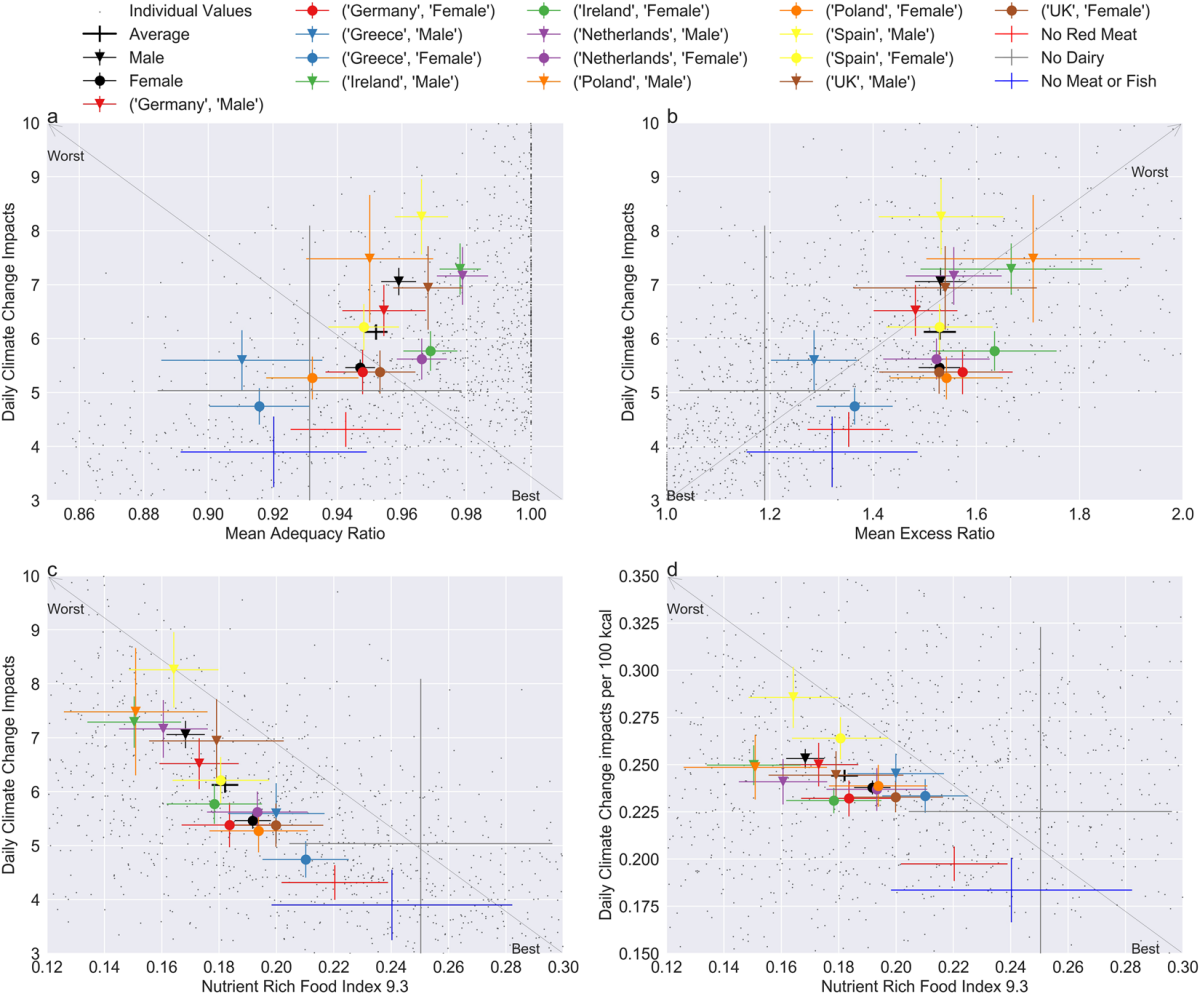
Relationships between impacts and nutrition. (**a**–**c**) show average daily climate change impacts (kgCO_2_eq) on the y-axis. (**d**) shows the climate change impacts per 100 kcal. Nutrition indicators (x-axis): (**a**) MAR, (**b**) MER, and (**c**–**d**) NRF9.3. Each individual is marked by a gray point. Data points marked with a circle or triangle represent the female or male subset, respectively, and no marker indicates both males and females were considered for the average. Length of the error bars represent the 95% confidence interval for the standard error of the mean. Similar figures for biodiversity loss and water scarcity footprint are included in the Supplementary Figs S1 and S2. See Supplementary Table S1 for sample size numbers.

For each environmental impact category, a significant difference between some, but not all, of the gender, country, and dietary pattern subsets was found (Fig. 2 for climate change, Supplementary Fig. S1 for biodiversity loss, Fig. S2 for water scarcity footprint). Climate change impacts for women were always statistically lower than their corresponding country’s male cohort. To compensate for the observed higher kcal intake of men (2826 to 2350 for women, p-value = 4.3e-17), all individual’s impacts were adjusted to a 2000 kcal diet as described in Willett^34^, however even when adjusted, female impacts (except Greek and Dutch subsets) continued to be statistically lower than the male’s (Supplementary Table S1), confirming previous results of Meier^9^. This result is further reinforced when looking at Fig. 2d and Supplementary Fig. S1d, which shows that on average women had lower impacts per 100 kcal consumed compared to men. The average eating patterns of genders indicate that men eat significantly more (in terms of mass) than women in almost every food group (p-value = 1.9e-6), except fruits and vegetables. The fact that men eat more, especially in food groups with higher impacts per gram like meats and fats, drives their higher impacts when compared to women in most subsets. However, men did have a slightly higher average mean adequacy ratio nutrient intake (Fig. 2a) compared to women (0.96 to 0.95, p-value 0.001), meaning that while they did have higher impacts, they also were more likely to consume recommended amounts of nutrients. Diet quality defined using a nutrient intake efficiency indicator (Fig. 2c, Supplementary Figs S1c and S2c), however, shows that women have better quality diets than men, as they consume more nutrients through fewer calories.

Culturally, the largest impacts were seen in the Spanish subsets (Fig. 2, Supplementary Figs S1, S2, and Table S1), driven by the 40% higher meat and fish consumption of this group compared to the average, as meat and fish have relatively high impacts per gram in all impact categories (Supplementary Figs S3–S13). For reference, all food group consumption rates per subset are in Supplementary Table S2. Dutch males also had a tendency for higher impacts (Supplementary Table S1) due to their higher than average energy consumption in most food groups except meat and fish when compared to sample average energy consumption. Their eating pattern allowed them to benefit from a higher than average mean adequacy ratio (MAR) while maintaining average mean excess ratio (MER) values. The Greek subsets had both a lower mean adequacy ratio and mean excess ratio (possibly a combination of their eating patterns and their lower kcal consumption, shown in Supplementary Fig. S15). This combination of lower kcal consumption and lower mean excess ratio drove up Greece’s nutrient intake efficiency (NRF9.3) compared to other countries, especially for the male subset (Fig. 2c). The impacts per 100 kcal (Fig. 2d, Supplementary Figs S1d and S2d) shows that the calories consumed by the Spanish population, regardless of gender, tend to be high in all impact categories. Most other countries (except a lower water scarcity footprint per 100 kcal for the Irish male subset and a lower biodiversity impact per 100 kcal for both UK subsets [Supplementary Fig. S2d]), however, were not significantly different from the average impact per 100 kcal. This indicates that although countries have widely varying impacts, this is due not only to what they are eating, but also to how much they are eating, as we saw above. We see for impacts and mean adequacy ratios there are similarities more towards countries rather than genders, and for nutrient intake efficiencies similarities lean towards genders. This implies that in order to improve diets, differences among not only the country’s eating patterns should be addressed, but also differences between genders in the countries, especially for countries with large observed gender differences in eating patterns like Poland and Spain.

Diets with no red meat or vegetarian diets (defined as no meat or fish) have considerably lower environmental impacts (Fig. 2, Supplementary Figs S1 and S2) and impacts per 100 kcal (Fig. 2d, Supplementary Figs S1d and S1d) than all other subsets in all impact categories. Based on the reported results, they did have nearly double the average intake of legumes (p-value 0.0003 compared to reported average of the entire sample), which are often considered a substitute for meat and which can have a high biodiversity impact (Supplementary Fig. S3) depending on their country of production (i.e. soybeans from Brazil). However, the increased impacts of legumes did not offset the lower impacts achieved through reduced meat consumption. Even with the lower impacts of no red meat diets, the average mean adequacy ratio (0.94) was statistically indistinguishable from the population average (0.95, p-value 0.21). For vegetarian diets (average mean adequacy ratio of 0.92), there was a statistically significant difference from the population average (p-value 0.025). The no red meat and no dairy diets were the only subsets to maintain both an average mean adequate nutrient ratio in combination with a lower than average mean excess ratio.

In Fig. 2 we see that on an individual level there were loose linear correlations with climate change impacts as MAR (r^2^ = 0.23) or MER (r^2^ = 0.54) increased, and as NRF9.3 (r^2^ = 0.53) decreased. However, the variability of impacts was large. For example, for people with a perfect MAR (1.0), impacts ranged from approximately 4 to 20 kgCO_2_eq per day. Most individuals (77%) with a perfect MAR had higher than average impacts. However, the individuals with both a perfect MAR and lower than average impacts tended to consume much less meat, dairy, and sweets compared to their above average impact, perfect MAR counterparts. Genders were represented nearly equally along the perfect MAR distribution (49% males, 51% females), but country distribution was not as even. Among the seven countries, Greece was under-represented with only 7% of the sample reaching a perfect MAR and Ireland and the Netherlands were overrepresented at 22% and 19%, respectively. A higher impact of a certain subset does not necessarily mean better mean adequacy ratios, and similarly a lower adequacy ratio does not always translate to lower impacts. For example, the higher than average impacts observed by both Spanish subsets did not yield a higher than average MAR value, whereas the higher MAR values observed by Irish subsets did not mean statistically higher impacts. These cultural and gender aspects influence both the impacts and diet quality of individuals, meaning that diets have the potential to be improved simultaneously for better diet quality and lower impacts by altering eating patterns.

When deciding what nutrition indicator to use to quantify the diet quality of an individual, it is important to consider all aspects. The use of NRF9.3 as a measure of a high quality diet would deliver the assumption that Greek people had a much healthier than average diet. This misses the fact that people were consuming fewer than recommended nutrients in the Greek subsample. The severity of health impacts from this under-consumption (or overconsumption with regards to the mean excess ratio) are out of the scope of this paper, however low dietary fiber, fruit, and whole grain intake has been ranked high in terms of disability adjusted life years by the Global Burden of Disease^28^, indicating that this nutrient and these food types may play a vital role in people’s health. Other dietary patterns, such as high processed meat or sodium intake, can be directly related to health impacts such as high cholesterol, high blood pressure or obesity, among other diseases, and heavily contribute to the worldwide DALYs, particularly in developed countries.

### Best Practice Diet

Good quality diets, as defined by a combination of high mean adequacy ratios (MAR) and low mean excess ratios (MER) or as a high nutrient efficiency ratios (NRF9.3), had lower than average impacts (Supplementary Table S1). The combination of high mean adequacy ratios and low mean excess ratios occurred for only 1.9% of the women and 0.8% of the men using the adequate intake values shown in Supplementary Table S4. Poor quality diets, defined as having either high mean excess ratios or low nutrient efficiency ratios, had above average amounts of food consumed in every food group, and because of the tendency for overconsumption (average kcal 3566 ± 1147 [SD]), these individuals had higher than average impacts in all impact categories. A ‘best practice diet’ was determined based on the average eating pattern of individuals who not only met the good quality diet criteria of a high MAR and low MER, but also fell in the lower third of impacts in all three impact categories (<0.5% of the individuals). Their daily average impacts were water scarcity footprint: 191 liteq, biodiversity: 2.55E-14 PDF*yr, and climate change: 4.2 kgCO_2_eq, but they also had a relatively low energy intake at 1949 (±382 [SD]) kcal. In this assessment, environmental impact categories were weighted equally to determine which diets were considered low impact when all three impact categories were considered together. However, changing the weighting of the environmental impact category in characterizing low impact diets can prioritize consumption of different food groups. Supplementary Fig. S14 shows the observed eating patterns associated with various diet combinations and impact categories.

Eating patterns of the various country and gender subsets were compared to the best practice diet defined above. Every subset required some changes in their eating patterns (Supplementary Table S3) to achieve a diet with the combination of low impacts, a high mean adequate ratio, and a low mean excess ratio. From the average eating patterns, a decrease in the amount of meat (−42%) (as has been recommended elsewhere^11^), sweets (−60%), fats (−66%), and drinks (−37%) consumption, and an increase in vegetables (+60%), and cereals (+65%) consumption was necessary, however there were large variations between the different subsets. For example, to achieve the best practice diet, the Greek female subsample should increase dairy consumption by nearly 15%, while the Polish male subsample should decrease it by 42%. For nearly all subsets (except Irish females), the intake of the cereal food group should be increased significantly. For reference, a Supplementary Table S3 shows the required eating pattern changes to achieve those of the best practice diet for each subset. Table 1 shows the potential impact reductions from changing from the average subset’s diet to the best practice diet. Extrapolating these reductions to the entire respective countries, the largest possible environmental impact reductions are possible in Germany, Spain, and the UK because of the large populations. The largest possible individual reductions are observed in the Spanish, Dutch, and Irish subsamples.

**Table 1:** Potential daily reduction in environmental impacts due to dietary changes from the average subset’s diet to the best practice diet for individuals and extrapolation of these reductions to the country’s entire population based on World Bank 2010 population statistics.

		Germany	Greece	Ireland	Nether-lands	Poland	Spain	UK
	population	81780000	11150000	4560000	16620000	38180000	47080000	62770000
Daily kgCO_2_eq	person	1.7	0.9	2.2	2.2	1.7	3.0	1.7
	country	1.4E + 8	9.99E + 6	9.9E + 6	3.65E + 7	6.58E + 7	1.43E + 8	1.06E + 8
Daily liteq	person	68.0	41.1	79.5	102.4	59.9	112.0	68.8
	country	5.56E + 9	4.58E + 8	3.62E + 8	1.70E + 9	2.29E + 9	5.27E + 9	4.32E + 9
Daily PDF*yr	person	1.92E-14	1.91E-14	2.30E-14	2.70E-14	2.04E-14	3.47E-14	1.63E-14
	country	1.57E-6	2.13E-7	1.05E-7	4.49E-7	7.79E-7	1.63E-6	1.03E-6

While the analysis above represents average recommendations, there was a large spread between individuals in both nutrient intake and impacts associated with diets. Personalized recommendations would therefore vary between individuals depending on current eating habits, and personal factors such as an individual’s age, gender, physical activity level, or health.

### Limitations of the study

There are some limitations to this analysis. Where possible, sensitivity analyses were conducted to quantify the effects of these limitations on the final results.

The results of this analysis are dependent on reported habitual intake from individuals participating in the Food4Me study as well as the chosen recommended nutrient intake values. Therefore, the uncertainties that are associated with dietary recall studies^38^, such as under or over reporting of food intake, or biases of participants to overestimate consumption of healthy foods, are also relevant to this paper. In addition, the consumption of vitamin and mineral supplements was not considered in this paper, meaning that MAR values shown here may overestimate nutrient deficiencies for individuals that consumed supplements in addition to their diet. The nutrition indicators used here were based on the European Food Safety Authority’s (EFSA) intake recommendations^39^, which can be different than country specific recommendations^40–42^, however they represent a European wide recommendation and as such are applicable to the data being considered here. To examine the impact of altering nutrient recommendations a sensitivity analysis was done with the US National Institute of Medicine^43^ intake recommendations (Supplementary Tables S4 and S5). It was found that there was an average decrease in the mean adequacy ratio (MAR value) of 4.5% when using the US National Institute of Medicine recommendations instead of the EFSA recommendations. MER was most sensitive to saturated fat, and removing this from the calculation decreased average MER values by 13%. Therefore the results are not expected to largely vary with nutrient recommendation changes. In addition, while under or over consumption of certain nutrients have directly been linked with risk of certain diseases^28,44^, nutrient intake may not directly correlate to an individual’s health due to factors such as genetics or severity of under or over consumption, which were not considered here.

An advantage to this study is that data was collected in an identical manner across the entire sample population, food consumption and nutrient intake results were calculated using identical methods, and under and over consumers were eliminated using the same factors, allowing comparisons across the study population. Regardless, kcal intake from other nutrition studies^45–50^ and from FAO kcal availability per country^51^ has been analyzed for comparison (Supplementary Fig. S15). As other country specific studies each may use differing methods of data collection and under/over reporter corrections, it is difficult to offer a direct comparison to kcal intake under the Food4Me study, however, the food frequency questionnaire (FFQ) used in the Food4Me study has been validated against another existing and widely used FFQ and against weighted food records and has been determined to be an acceptable method of collecting dietary information^38,52^. When comparing the Food4Me kcal consumption to FAO available kcal data, available kcal are higher than consumed kcal. This is expected, as available kcal does not necessarily mean consumption, and may simply be wasted food. In addition, the sample population partaking in the Food4Me study may not reflect food consumption patterns of the country as a whole, as a sample size of approximately 200 is not representative of an entire country. Characteristics of the participants have been analyzed and are presented in Livingstone, K.M.^31^.

Environmental impacts associated with food waste were not included in the above assessment, as food waste was not reported in the survey. As different food groups have different food wastage rates^53^, a sensitivity analysis was done to determine the effects of including food waste impacts on an individual’s climate change impact for three diet types with varying food group consumption patterns (described in Methods). Including average food waste rates increased the median impact of Diet 1 (high meat intake) by 19%, Diet 2 (high plant product intake) by 22%, and Diet 3 (high dairy intake) by 18%. Given the relatively similar changes in impacts observed between the three diet extremes, it is unlikely that including food waste in this assessment will significantly alter the relationship between impacts and different eating patterns of the subsamples investigated here.

This study assessed the environmental impacts in a regionalized manner, but then calculated production weighted globalized averages to evaluate the environmental impacts of each individual’s diet. This was done to provide identical baseline data on which diet quality could be compared among different individuals. An individual’s food purchasing habits, however, can cause variation in their impacts. For example, the impacts may change from varying animal farming methods^54^, methods of crop growth (e.g. utilization of greenhouses and influence of seasonality)^55,56^, and location, particularly for water scarcity footprint and biodiversity loss^21,57^. These differences were intentionally disregarded here to allow us to assess the influence of dietary patterns, but a sensitivity analysis of the effects of various purchasing decisions was completed. The resulting impact ranges are included in the Supplementary Electronic Table. For example, assuming low and high impact vegetable production (with and without greenhouse heating) causes an average change in an individual’s total impacts of −2.4% and +1.2%, respectively. By contrast, low versus high impact meat production had a much larger influence, with average observed changes of −12.8% and +9.0%, respectively. When evaluated on a country specific gender subset basis (Supplementary Fig. S16), it can be seen that because of eating patterns, the percent change in certain subset’s impacts are influenced more than others. For example, changes in beef production methods can significantly affect the change in Spanish subset’s climate change impacts, but has no significant influence on the Polish subset’s impacts.

## Conclusions

While impact reduction possibilities are more limited with people already eating good quality diets, they are still possible through further reduced consumption of certain foods, particularly meats, sweets, and drinks, in combination with increases in vegetables and cereals. Individuals with poor quality diets tend to be the highest consumers in most food groups, and therefore have much higher impacts. Reducing food consumption in most food groups would benefit them not only in achieving a healthier diet, but also in reducing their impacts. However, in combination with certain food group reductions, they should increase their consumption of fruits, vegetables, and cereals to achieve a low impact, good quality diet. Food reduction, particularly for the people already exhibiting a good quality diet, needs to be done with care, as their kcal intake is already relatively low, and intake levels below a threshold of around 2000 kcal trend towards nutrient deficiencies. For the average population studied here to move towards a low impact, good quality diet, vegetable and cereal consumption should be increased, and meat, drinks, and sweets should be reduced, however in the end, recommendations should be made on an individual level rather than as an average.

This study is unique in that it allows for the comparison of self-selected and reported diets between individuals in seven European countries with varying cultures. Identical food data collection methods, nutrient consumption evaluation methods, and impact assessment system boundaries were used for each individual, allowing for a cross-cultural comparison of impacts between genders, countries, dietary patterns, and diet quality. The analysis shows definite cultural and gender factors influencing both the impacts and diet quality of individuals through differing dietary patterns, meaning that diets have the potential to be improved simultaneously for better diet quality and lower impacts by altering eating patterns. This information allows for governments, health organizations, and consumers to cater to particular subsets when designing food related programs focused on reducing their contributions to environmental impacts while improving their health.

## Methods

Food consumption data was obtained from the Food4Me study^58^ and measured through the use of an online food frequency questionnaire (FFQ). The previous month’s habitual food consumption was assessed by collected data on consumption frequency and portion size for 162 food and drink items^38,59^, and included over 1400 men and women from seven European countries (Germany, Greece, Ireland, Netherlands, Spain, Poland, and the UK) between the ages of 18 and 79, with full details regarding age, gender, weight, health, physical activity levels, and reasons for participating in the study, published elsewhere^31^.

Daily nutrient intake values were based on the European Food Safety Authority’s (EFSA) adequate intake (AI) dietary reference values (Supplementary Table S4)^39^, which are based on experimental data and are the recommended average daily nutrient intake levels for healthy individuals^43^. The diet quality of an individual was measured using two absolute indicators and one efficiency indicator. The first absolute indicator, mean adequacy ratio (MAR), has been developed as a measure of adequate nutrient consumption^40^ for 19 nutrients. This value correlates with nutrient deficiencies, with calculation details in the Supplementary Section S6. Because mean adequacy ratio does not capture consumption of nutrients that should be consumed in limited quantities, the mean excess ratio (MER), as developed by Vieux *et al*.^18^ was also calculated (details in Supplementary Section S6), and includes the average consumption ratios of saturated fats, sugars, and sodium. The Nutrient Rich Food Index 9.3 (NRF9.3) was used as an efficiency indicator to measure the nutritional quality of each diet and includes the combination of both beneficial and harmful nutrients as well as energy intake. This was developed as a method of ranking the nutritional quality of foods and was found to be highly correlated to diet quality as measured through the Healthy Eating Index (HEI)^25^. Details of NRF9.3 calculation methods are included in the Supplementary Section S6.

Impact values per gram of food were calculated for each of the 162 foods/dishes on the FFQ, with composite foods broken down into their three main ingredients by mass using a generic recipe or product label. In many cases, impacts were available per crop type or ingredient (e.g. tomatoes) but not for a product (e.g. ketchup) derived from that crop. In this case, the impact associated with the root product (tomatoes) was determined and conversion factors, as provided in Scherer *et al*.^12^ were used to calculate the impact of the derived product. The Supplementary Electronic Table shows the foods/dishes, their three main ingredients, conversion factors, associated processing energy and references (included only for climate change), and any assumptions.

The impact of each gram of food was calculated for climate change, water scarcity footprint, and land-use driven biodiversity loss using a combination of food production inventory databases^60,61^. IPCC Global Warming Potentials (GWP) 2013 100 years characterization factors^62^ were used to calculate climate change impacts utilizing Brightway^63^. The GWP were based on the radiative efficiency of greenhouse gases relative to carbon dioxide over a 100 year time span^62^. The water scarcity footprint, measured as liters equivalent (liteq) per gram of food, was calculated by multiplying a monthly, regional water stress index^57^ with crop specific irrigation requirements to determine the global production-weighted water footprint per crop. The land-use driven biodiversity impacts quantified the percentage of global species that are lost at steady-state (using final units of global potentially disappeared fractions (PDF)*years), i.e. it is an indicator of global extinctions that will result in the long-term as a consequence of land use, developed in Chaudhary *et al*.^21^. In both the water scarcity footprint and the biodiversity assessments, global weighted production averages were used, regardless of the country of consumption, to allow for an assessment of the impact due to varying diets and not to the changes in the supply chain. Water scarcity footprint and land-use biodiversity loss impacts associated with livestock products (beef, chicken, milk, eggs, pig, sheep, and fish) were calculated based on both the cultivation of animal feed required per gram of product as well as required pasture area^64,65^, however biodiversity impacts due to fishing were not considered due to a lack of life cycle impact assessment methodology for aquatic biodiversity loss, therefore these impacts will be underestimated. For each environmental indicator, each individual’s impacts were calculated by multiplying the impacts per gram of each of the 162 foods/dishes (Supplementary Figs S3 through S13 and Electronic Table) by the reported daily grams of the corresponding food consumed by that person. An environmental impact efficiency indicator, calculated as the ratio of impacts to energy intake, was also determined for each individual. This indicator shows the impacts associated with an individual’s kcal consumption, regardless of the nutrients consumed, and can show whether primarily high impact or low impacts foods are consumed in relation to their energy intake.

A best practice diet was constructed based on the eating patterns at the intersection of low environmental impact and good quality diets. Low environmental impact diets were defined as diets falling in the lower third for all environmental impact categories (climate change, biodiversity loss, and water scarcity footprint). It is recognized that there are various ways of defining good quality diets^66^, however to identify the recommended eating pattern in this study, they were defined as diets with a combination of high MAR (upper third of the individuals) and low MER (lowest third of the individuals), based on studies linking these indicators to healthy diets and based on a similar analysis by Vieux *et al*.^18^. Poor quality diets were defined as diets either having a high mean excess ratio (highest third of the population) or a low nutrient efficiency score (NRF9.3) (lowest third of the population). A true poor quality diet would be a combination of high MER and low MAR values, however this combination was not found amongst the sample population, due to the fact that as MER increased, MAR tended to as well (Fig. 1).

The food waste sensitivity analysis compared original impacts to impacts including food waste. A study based on Swiss data has recently found that for plant based products, avoidable losses in food waste accounts for an additional 54% of impacts associated with the product’s final intake, whereas for meat or dairy based products this rate is lower, at 27% and 20%, respectively^53^. Food waste was not considered for processed food items such as drinks, fats, sweets, soups, and sauces. Three observed diet types were isolated from the sample - Diet 1: those consuming high meat (upper third of the population) and low dairy and plant based products (lower third), Diet 2: those with high plant based products and low dairy and meat intake, and Diet 3: those with high dairy and low meat and plant based products. The original impacts of individuals falling in these diet types were compared to their impacts when including food waste for each food group to determine the effects of including food group specific food wastage rates.

A sensitivity analysis was performed to compare the effects of using global and regional environmental impacts. Individual impacts were calculated for four additional food production scenarios: low vegetable impacts, high vegetable impacts, low beef impacts, and high beef impacts. The percent change in total daily impact from global to each scenario was calculated for each person and average change in impact for each scenario was determined. Then the average percent change for each country specific gender subset was calculated under the different scenarios. Significance of the observed change in impacts was determined by comparing the scenario specific impacts of each subset to the global impacts of each subset.

Confidence interval upper and lower boundaries for each population subset represent the upper and lower 95% limit (z-value of ±1.96) of the standard error of the mean (SEM). SEM was used instead of standard deviations to better visualize the significance of the difference of the means between sample subset populations, however statistical significance between subsets was verified using an unpaired two sided t-test under the assumption that p-values lower than 0.05 indicated statistically significant differences between the means of subsets.

All data generated or analyzed during this study are included in this published article (and its Supplementary Information files) except raw FFQ data, which is not publically available.

## Electronic supplementary material

Supplementary Information

Supplementary Information
