# Supplementary material for: Comparison of Environmental Impact and Nutritional Quality among a European Sample Population – findings from the Food4Me study
Source: Sci Rep. 2018 Feb 5;8:2330. doi: 10.1038/s41598-018-20391-4 (PMC5799208; doi:10.1038/s41598-018-20391-4)
Supplement: Supplementary file 1 — Supplementary Information [file 41598_2018_20391_MOESM1_ESM.pdf]

Supplemental Information

Comparison of Environmental Impact and Nutritional Quality among a European Sample Population – findings from the Food4me study

Christie Walker[1,*], Eileen R. Gibney[2], Stefanie Hellweg[1]

[1] ETH Zurich, Institute of Environmental Engineering, 8093 Zürich, Switzerland

[2] University College Dublin, Institute of Food and Health, Dublin 4, Ireland

[*] Corresponding author: Email: walker@ifu.baug.ethz.ch Phone: +41 44 633 7145

Table of Contents

S1. Supplementary Environmental Impact Results
S2. Supplementary Results of Each of the Foods/Dishes Analyzed
S3. Supplementary Determination of Recommended Diets Results
S4. Supplementary Comparison of Energy Intakes
S5. Supplementary Scenario Analysis for Different Food Production Methods
S6. Supplementary Material for Data Sources, Nutrition, and Environmental Indicators

S1. Supplementary Environmental Impact Results

Table S1. Daily average impacts for each category and population subset. Ranges represent the standard deviation of the subset. Severity of the impact in relation to other subsets is represented through shading. A darker shade indicates relatively higher impacts while a lighter shade indicates relatively lower impacts. Italicized numbers indicate a significant difference between the male and female subsets of the specific country, or in the case of no meat and fish, no red meat, no dairy, or diet quality indicators, diets with significant differences from the TOTAL population average. Statistical significance between subsets was verified using an unpaired two sided t-test under the assumption that p-values lower than 0.05 indicated statistically significant differences between the means of subsets.

		As consumed						Adjusted to 2‘000 kcal					
		Climate Change		WFP		Biodiversity		Climate		WFP		Biodiversity	
	n=	$kgCO_2eq$		liters eq		PDFyr*10E-13		$kgCO_2eq$		liters eq		PDFyr*10E-13	
TOTAL	1457	6.14	±2.78	267.57	±107.63	48.44	±28.5	4.99	±1.62	224.22	±65.71	39.49	±22.42
Male	602	*7.08*	*±3.18*	*295.02*	*±119.91*	*56.29*	*±34.53*	*5.34*	*±1.9*	*229.56*	*±72.97*	42.77	±27.44
Female	855	*5.48*	*±2.24*	*248.25*	*±93.42*	*42.91*	*±21.73*	*4.75*	*±1.34*	*220.46*	*±59.83*	37.18	±17.73
COUNTRY													
German													
Male	95	*6.54*	*±2.35*	271.32	±96.65	*48.64*	*±24.13*	*5.2*	*±1.53*	220.44	±58.47	38.14	±19.25
Female	110	*5.4*	*±2.21*	248.77	±87.72	*41.37*	*±20.46*	*4.69*	*±1.31*	221.68	±60.71	35.78	±18.62
Greek													
Male	85	*5.61*	*±2.65*	248.78	±127.89	*49.36*	*±29.16*	4.97	±1.37	224.39	±73.55	44.32	±20.71
Female	123	*4.76*	*±1.91*	220.91	±78.89	*41.36*	*±22.81*	4.6	±1.33	214.77	±45.15	40.1	±18.33
Irish													
Male	84	*7.3*	*±2.23*	*289.24*	*±91.44*	*56.38*	*±19.73*	*5.19*	*±1.68*	209.17	±74.18	*39.85*	*±20.35*
Female	130	*5.79*	*±2.14*	*258.64*	*±84.15*	*43.4*	*±19.3*	*4.62*	*±1.02*	214.32	±58.09	*34.25*	*±14.96*
Dutch													
Male	109	*7.18*	*±2.84*	*322.92*	*±110.43*	*59.33*	*±36.27*	5.07	±1.95	242.85	±74.9	42.8	±30.61
Female	109	*5.64*	*±2.03*	*264.25*	*±91.07*	*45.64*	*±21.21*	4.73	±1.4	230.07	±63.35	38.59	±17.8
Polish													
Male	59	*7.49*	*±4.63*	*297.84*	*±158.93*	*58.09*	*±53.86*	*5.4*	*±2.51*	218.89	±78.14	41.79	±37.68
Female	142	*5.29*	*±2.42*	*231.62*	*±92.11*	*40.82*	*±24.01*	*4.69*	*±1.64*	209.09	±60.68	36.17	±21.48
Spanish													
Male	106	*8.28*	*±3.68*	*330.33*	*±125.27*	*70.16*	*±41.05*	*6.3*	*±2.25*	255.8	±76.3	*54.78*	*±33.88*

Female	106	6.22	±2.25	276.04	±112.1	50.14	±19.96	5.34	±1.38	242.67	±70.1	43.25	±15.67
UK													
Male	64	6.96	±3.16	290.59	±113.37	46.88	±23.25	5.11	±1.4	220.44	±62.64	32.4	±16.88
Female	135	5.39	±2.37	245.46	±97.51	39.43	±22.04	4.65	±1.09	217.32	±55.42	33.62	±14.33
DIETARY PATTERNS													
No Meat/Fish	24	3.94	±1.65	215	±118.98	19.86	±10.67	3.62	±0.99	202.99	±83.47	17.38	±9.52
No Red	94	4.35	±1.6	224.01	±92.5	21.87	±9.02	3.85	±1.00	205.24	±63.06	17.99	±9.84
No Dairy	7	5.09	±4.1	288	±165.4	16.9	±8.74	4.95	±3.38	282.32	±138.3	16.58	±7.30
DIET QUALITY													
High MAR/ Low MER	48	5.08	±0.99	238	±40.38	33.83	±14.85	Good Quality Diets					
High NRF9.3	481	4.24	±1.15	196.44	±52	32.23	±12.46						
Good Quality	19	5.09	±1.03	238	±42	34.01	±15.63						
High MER	481	8.39	±3.18	358.6	±114.5	67.91	±35.41	Poor Quality Diets					
Low NRF9.3	481	8.44	±3.24	357	±118	67.72	±36.32						
Poor Quality	435	8.6	±3.23	366	±116	69.33	±35.97						

Table S2. Average grams of food consumed for each of the subsets considered. Upper and lower limits are the 95% confidence intervals (z-value 1.96) of the standard error of the mean.

	Dairy		Fruits		Vegetables		Starches		Meat and Fish		Bread		Sweets		Drinks (divided by 10)		Soups and Sauces		Cereal		Eggs		Fats and Spreads	
German Male	306.65	±40.29	273.14	±48.41	182.08	±17.38	216.91	±19.81	171.6	±20.75	157.59	±21.61	97.23	±15.16	112.4	±13.48	71.85	±9.49	54.06	±14.7	25.04	±4.02	24.42	±3.84
German Female	347.07	±41.56	302.48	±47.55	209	±28.64	179.46	±16.58	121.64	±14.59	123.89	±20.02	102.93	±18.25	105.35	±10.49	78.02	±10.69	38.71	±10.71	23.73	±4.12	21.24	±3.14
Greek Male	281.64	±56.53	215.5	±44.49	191.45	±27.78	189.61	±25.78	200.25	±26.7	119.91	±24.49	90.67	±16.62	69.38	±12.44	47.07	±9.31	68.45	±22.2	32.88	±8.94	18.93	±3.6
Greek Female	250.08	±40.06	239.34	±31.13	214.38	±25.6	150.5	±16.44	160.35	±15.74	107.46	±38.78	90.49	±14.61	63.99	±11.64	48.4	±6.69	50.61	±11.38	22.5	±6.37	20.31	±3.08
Irish Male	324.7	±39.54	261.52	±65.03	197.47	±27.34	231.84	±22.58	225.23	±21.75	162.94	±30.25	140.99	±37.85	112.08	±12.84	98.78	±14.29	104.62	±19.79	54.34	±15.6	26.64	±5.65
Irish Female	346.82	±38.33	343.24	±51.5	267.32	±29.96	190.22	±16.6	180.53	±15.62	102.36	±13.81	130.96	±26.5	90.1	±9.48	112.42	±12.78	121.69	±21.4	37	±6.72	18.6	±2.49
Dutch Male	418.67	±58.7	292.61	±43.55	207.91	±25.48	214.99	±26.99	196.43	±36.18	252	±39.37	98.29	±14.45	131.88	±12.67	97.22	±13.39	89.8	±22.17	38.29	±11.96	23.67	±2.9
Dutch Female	345.78	±50.42	291.89	±37.64	203.96	±22.99	163.01	±13.34	135.31	±12.69	196.09	±30.69	87.5	±15.5	123.01	±10.85	103.27	±13.86	58.69	±17.06	20.59	±4.02	20	±2.55
Polish Male	495.14	±121.3	198.24	±42.19	166.81	±31.21	221.15	±35.88	246.53	±42.44	223.02	±53.56	125.32	±28.54	101.31	±13.77	129.07	±20.36	49.91	±22.88	43.13	±11.23	28.52	±6.96
Polish Female	343.58	±41.95	242.24	±34.39	194.91	±27.72	166.9	±22.29	156.34	±17.52	152.49	±31.38	108.11	±22.69	91.94	±8.64	117.61	±16.24	57.5	±13.0	29.48	±4.8	19.2	±3.11
Spanish Male	409.02	±67.73	258.7	±37.4	232.27	±25.53	207.69	±25.82	323.37	±31.28	165.1	±33.26	118.87	±21.35	91.66	±13.69	87.35	±12.85	38.12	±11.42	39.53	±6.35	16.26	±2.45
Spanish Female	415.11	±60.49	321.43	±58.51	214.86	±22.36	142.21	±17.3	233.42	±17.86	119.39	±26.64	96.14	±14.8	73.91	±7.83	82.99	±20.37	45.59	±15.97	34.7	±4.9	20.44	±4.2
UK Male	378.97	±59.36	304.08	±77.91	264.44	±34.34	248.62	±43.57	211.39	±23.96	113.9	±22.53	131.58	±41.21	112.3	±17.6	114.63	±22.77	109.45	±39.86	54.07	±17.66	15.6	±2.7
UK Female	353.72	±37.06	268.2	±37.95	249.03	±31.63	186.72	±18.11	164.38	±17.0	100.37	±21.79	108.34	±17.49	105.52	±10.79	94.93	±12.08	79.23	±14.63	35.12	±8.77	15.74	±2.34
Total	353.3	±14.06	274.73	±12.48	215.94	±7.54	188.99	±6.05	189.84	±6.46	146.45	±8.29	107.81	±5.85	98.12	±3.25	91.15	±3.94	71.11	±5.2	33.7	±2.21	20.32	±0.92
Male	369.67	±24.23	260.23	±19.33	206.32	±10.27	216.97	±10.57	226.06	±12.1	173.01	±13.09	112.8	±9.39	105.06	±5.41	89.75	±5.67	75.6	±8.88	39.83	±4.15	21.87	±1.54
Female	341.82	±16.81	284.97	±16.31	222.73	±10.61	169.28	±6.85	164.34	±6.45	127.8	±10.52	104.31	±7.46	93.24	±3.99	92.13	±5.4	68.15	±6.34	29.33	±2.33	19.23	±1.13
No Meat and Fish	308.47	±105.5	345.25	±171.1	347.15	±106.3	171.04	±38.14	0	±0.0	126.24	±38.94	89.98	±35.13	89	±21.81	79.01	±22.17	91.61	±37.32	37.39	±15.69	15.58	±7.23
No Red Meat	341.14	±55.31	365.19	±74.0	320.09	±42.24	168.86	±19.59	73.06	±15.35	117.85	±23.01	89.23	±16.74	101.97	±11.22	90.79	±15.74	115.06	±31.0	39.15	±8.47	18.87	±3.14

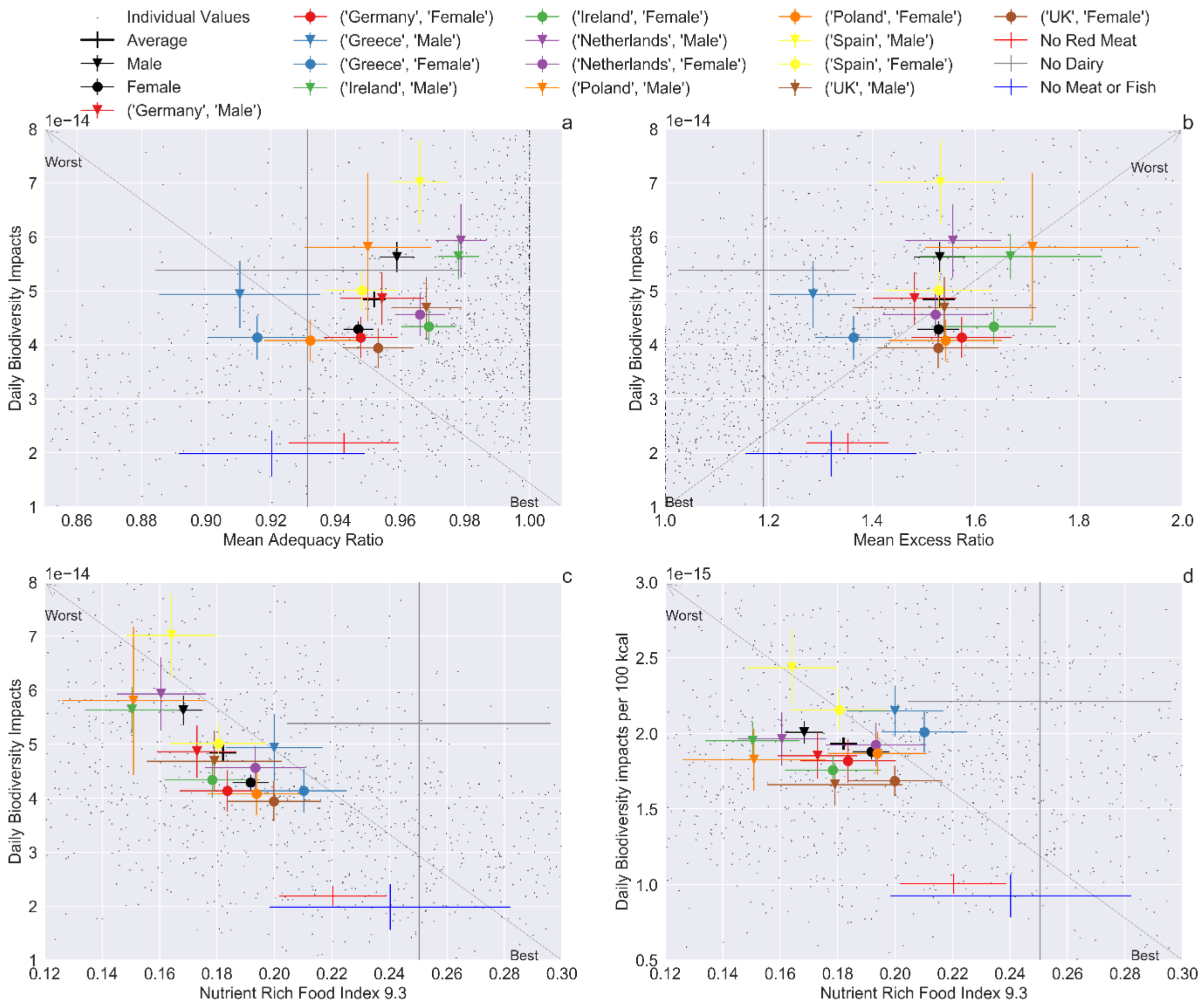

Fig. S1. Relationships between impacts and nutrition. a-c show average daily biodiversity impacts (PDF*yr) on the y-axis. d shows the environmental impacts per 100 kcal. Nutrition indicators (x-axis): a: MAR, b: MER, and c-d: NRF9.3. Each individual is marked by a gray point. Data points marked with a circle or triangle represent the female or male subset, respectively, and no marker indicates both males and females were considered for the average. Length of the error bars represent the 95% confidence interval for the standard error of the mean. See Table S1 for sample size numbers.

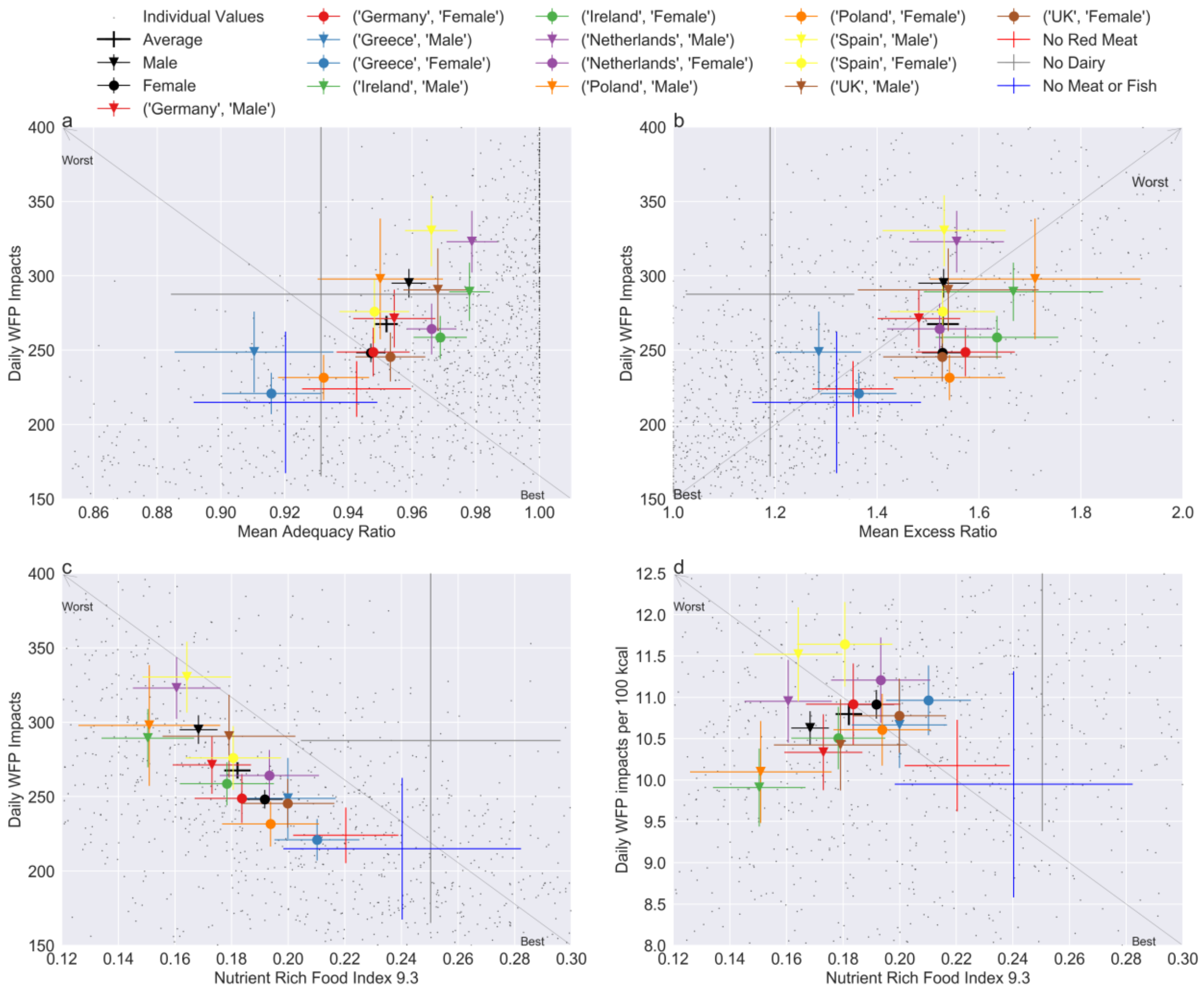

Fig. S2. Relationships between impacts and nutrition. a-c show average daily water scarcity footprint impacts (liteq) on the y-axis. d shows the environmental impacts per 100 kcal. Nutrition indicators (x-axis): a: MAR, b: MER, and c-d: NRF9.3. Each individual is marked by a gray point. Data points marked with a circle or triangle represent the female or male subset, respectively, and no marker indicates both males and females were considered for the average. Length of the error bars represent the 95% confidence interval for the standard error of the mean. See Table S1 for sample size numbers.

S2. Supplementary Results of Each of the Foods/Dishes Analyzed:

Impacts per gram for each food/dish in the various food groups for each impact category are shown in the figures below. Blue dots represent the average daily consumption (per person) of each food/dish. Dashed lines represent the weighted average (as consumed), and solid lines show the average regardless of consumption rates.

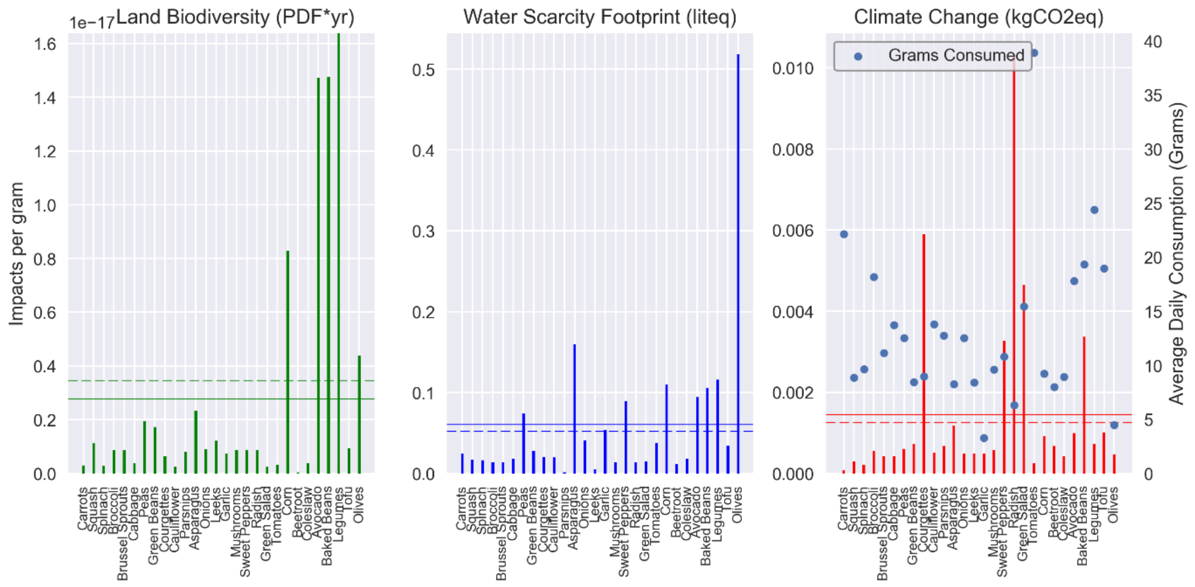

Fig. S3. Impacts per gram for each food/dish in the Vegetable food group for each impact category. Blue dots represent the average daily consumption (per person) of each food/dish. Dashed lines represent the weighted average, and solid lines show the average regardless of consumption rates.

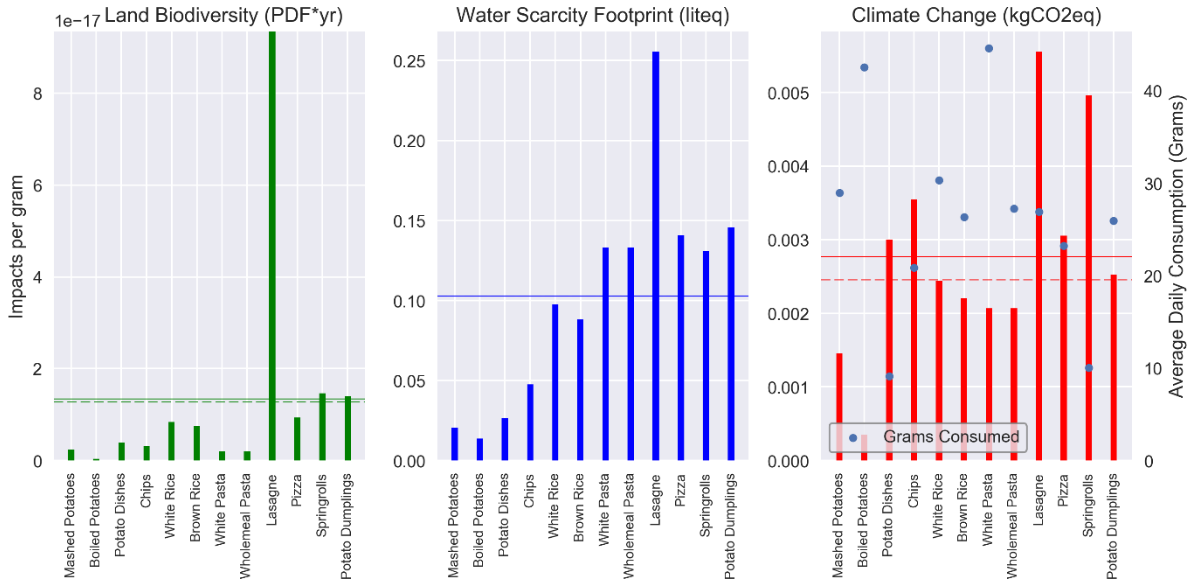

Fig. S4. Impacts per gram for each food/dish in the Potatoes, Rice, and Pasta food group for each impact category. Blue dots represent the average daily consumption (per person) of each food/dish. Dashed lines represent the weighted average, and solid lines show the average regardless of consumption rates.

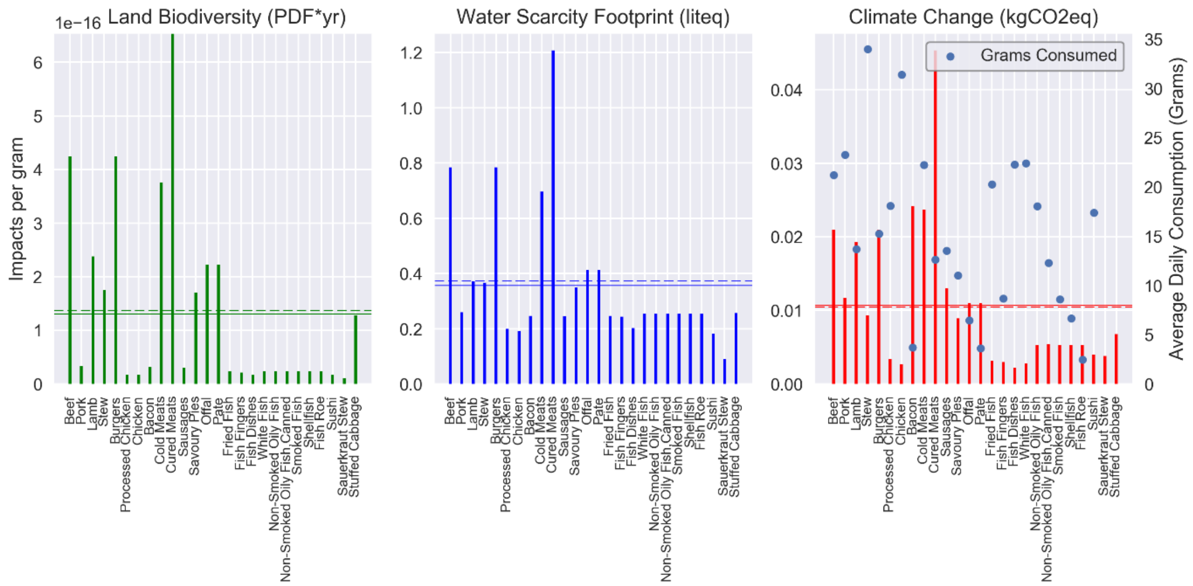

Fig. S5. Impacts per gram for each food/dish in the Meat and Fish food group for each impact category. Blue dots represent the average daily consumption (per person) of each food/dish. Dashed lines represent the weighted average, and solid lines show the average regardless of consumption rates.

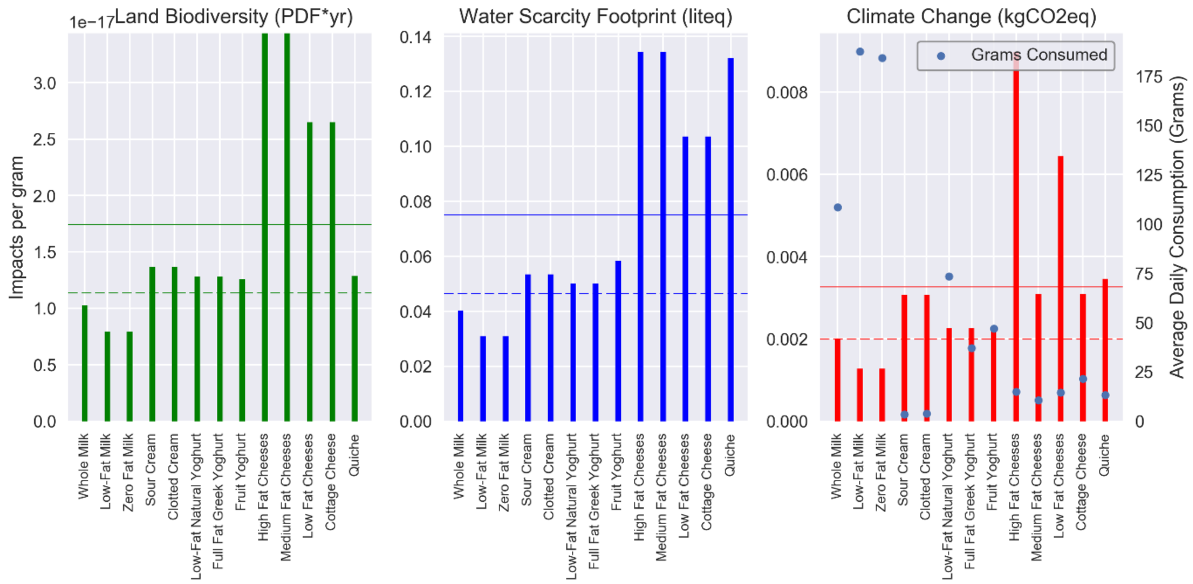

Fig. S6. Impacts per gram for each food/dish in the Dairy food group for each impact category. Blue dots represent the average daily consumption (per person) of each food/dish. Dashed lines represent the weighted average, and solid lines show the average regardless of consumption rates.

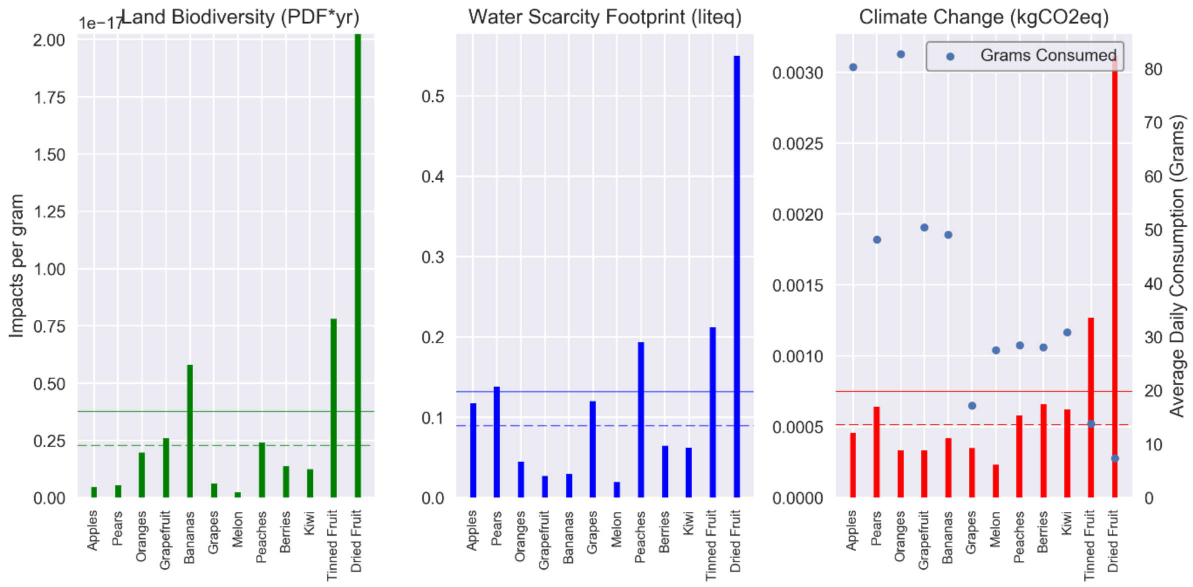

Fig. S7. Impacts per gram for each food/dish in the Fruit food group for each impact category. Blue dots represent the average daily consumption (per person) of each food/dish. Dashed lines represent the weighted average, and solid lines show the average regardless of consumption rates.

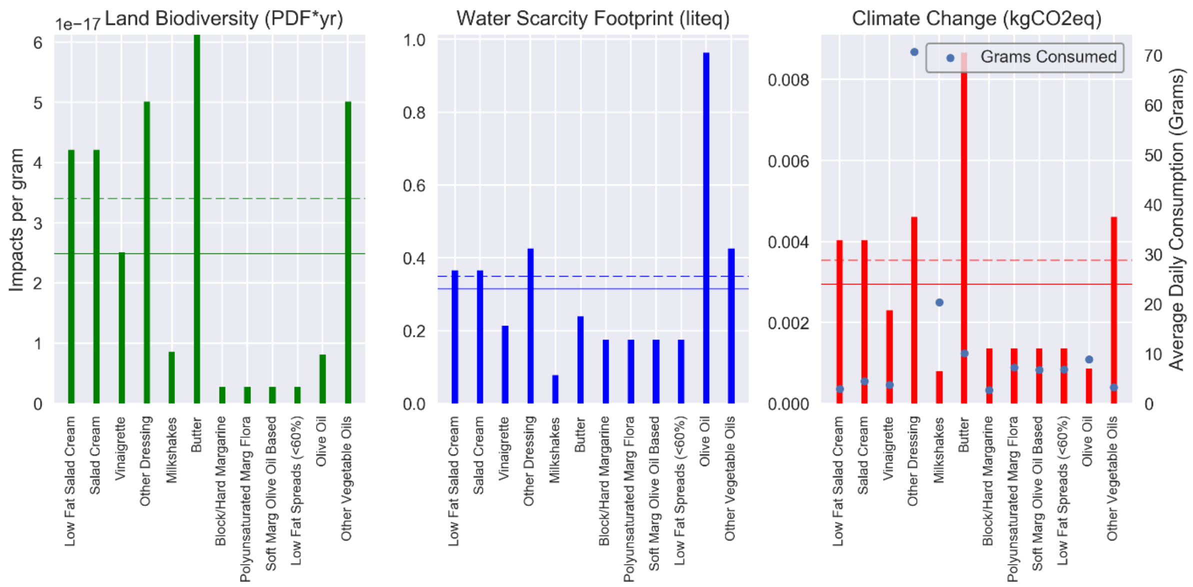

Fig. S8. Impacts per gram for each food/dish in Fats food group for each impact category. Blue dots represent the average daily consumption (per person) of each food/dish. Dashed lines represent the weighted average, and solid lines show the average regardless of consumption rates.

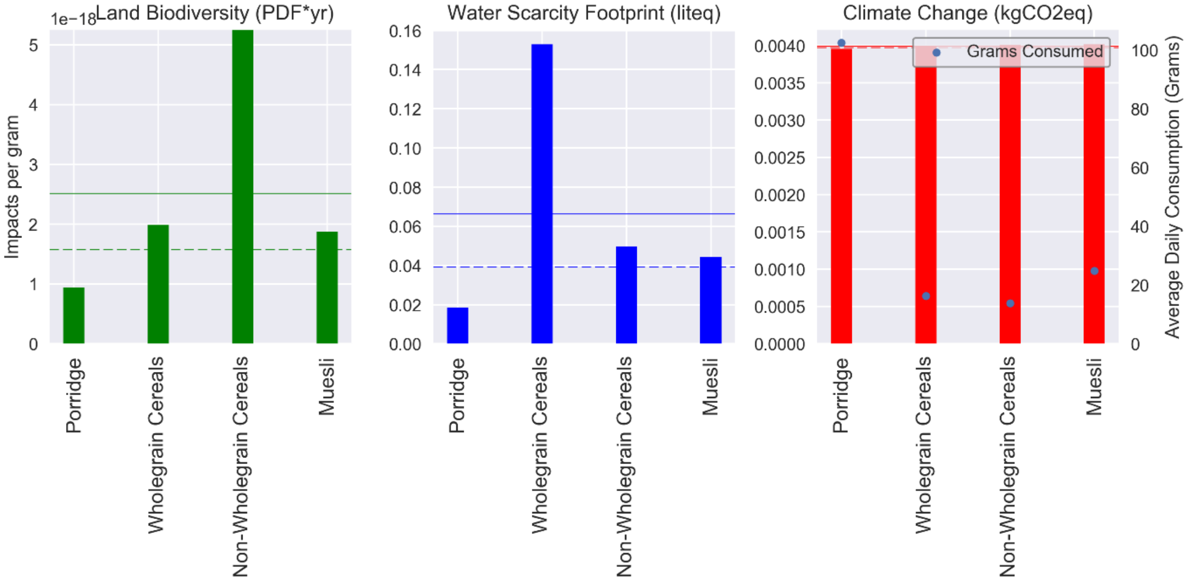

Fig. S9. Impacts per gram for each food/dish in Cereals food group for each impact category. Blue dots represent the average daily consumption (per person) of each food/dish. Dashed lines represent the weighted average, and solid lines show the average regardless of consumption rates.

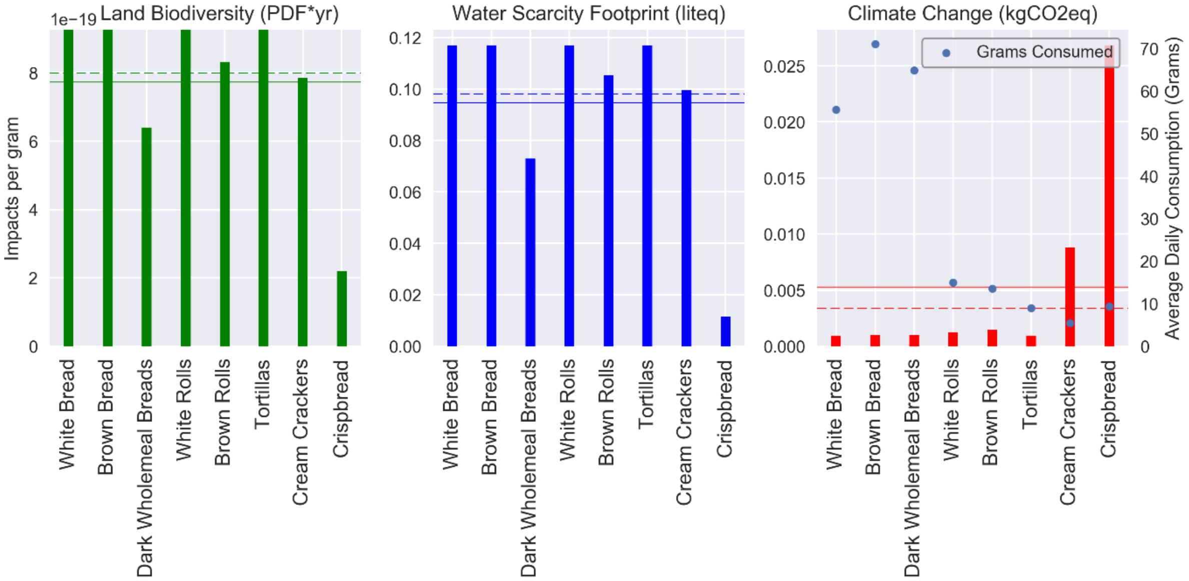

Fig. S10. Impacts per gram for each food/dish in Breads food group for each impact category. Blue dots represent the average daily consumption (per person) of each food/dish. Dashed lines represent the weighted average, and solid lines show the average regardless of consumption rates.

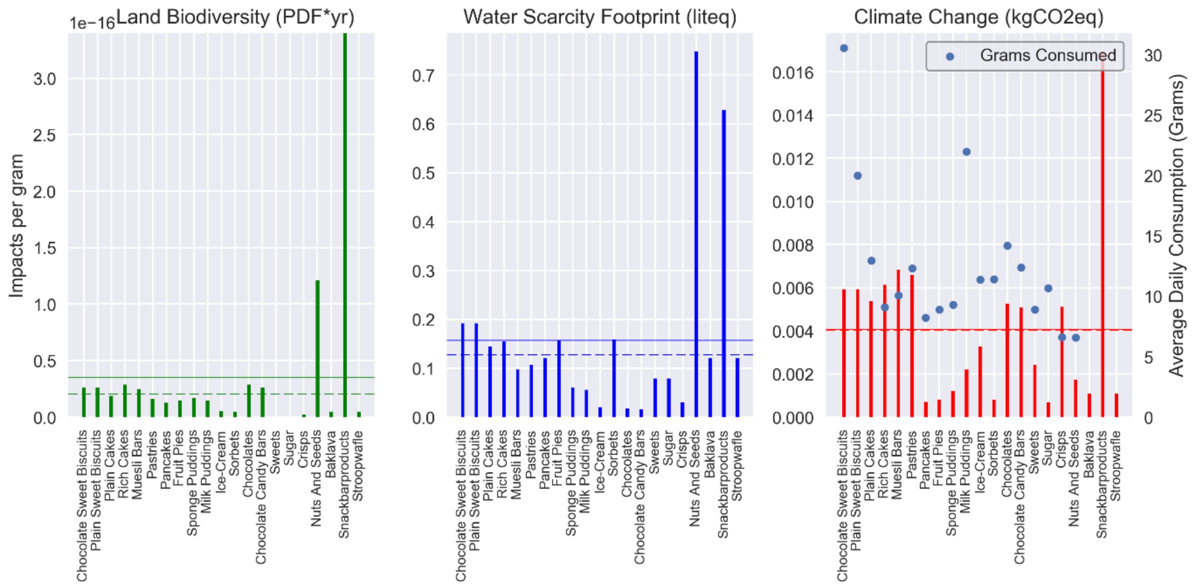

Fig. S11. Impacts per gram for each food/dish in Sweets food group for each impact category. Blue dots represent the average daily consumption (per person) of each food/dish. Dashed lines represent the weighted average, and solid lines show the average regardless of consumption rates.

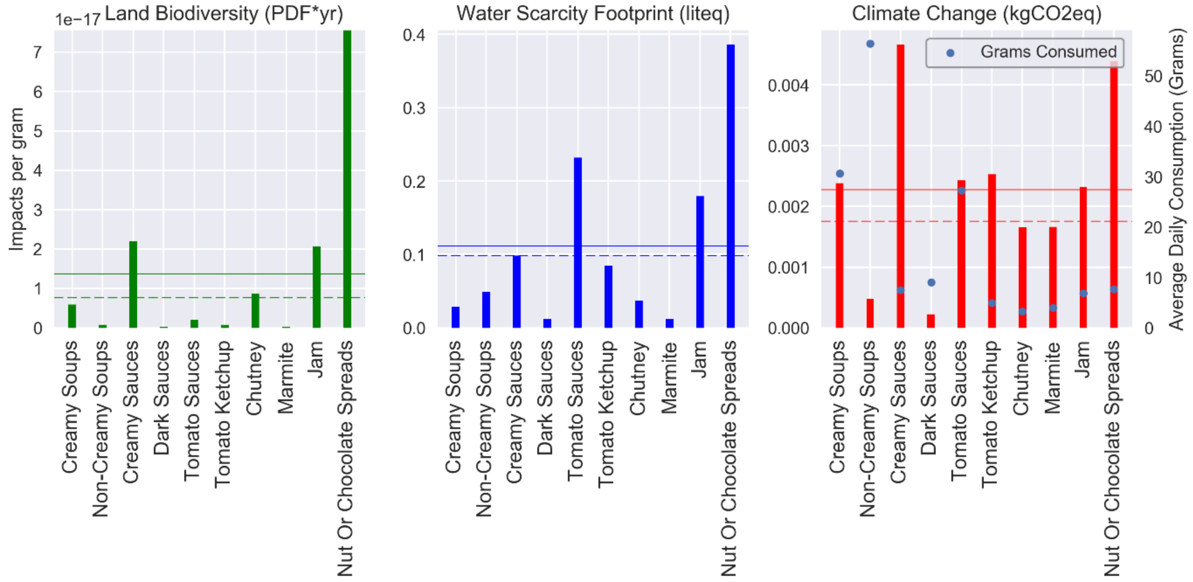

Fig. S12. Impacts per gram for each food/dish in Soups and Sauces food group for each impact category. Blue dots represent the average daily consumption (per person) of each food/dish. Dashed lines represent the weighted average, and solid lines show the average regardless of consumption rates.

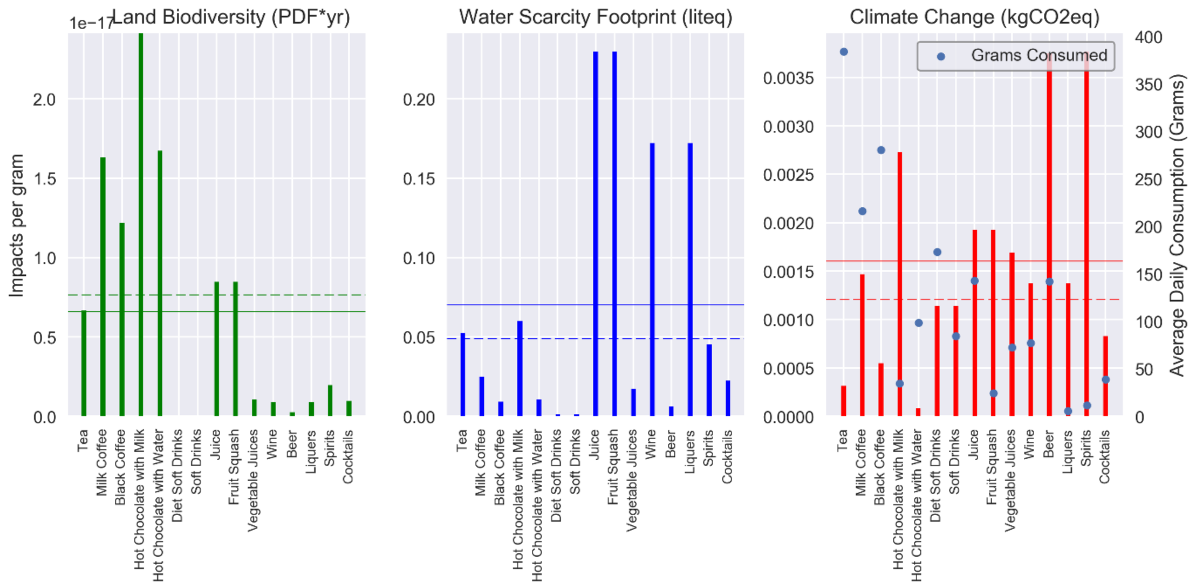

Fig. S13. Impacts per gram for each food/dish in Drinks food group for each impact category. Blue dots represent the average daily consumption (per person) of each food/dish. Dashed lines represent the weighted average, and solid lines show the average regardless of consumption rates.

S3. Supplementary Determination of Recommended Diets Results

We investigated what type of eating patterns (Fig 14) were associated with both good and poor quality diets (and the impacts associated with these diets (Table S1)), the eating patterns for low and high impact diets in each impact category, and the eating patterns that fell at the intersection of low impact and good quality diets.

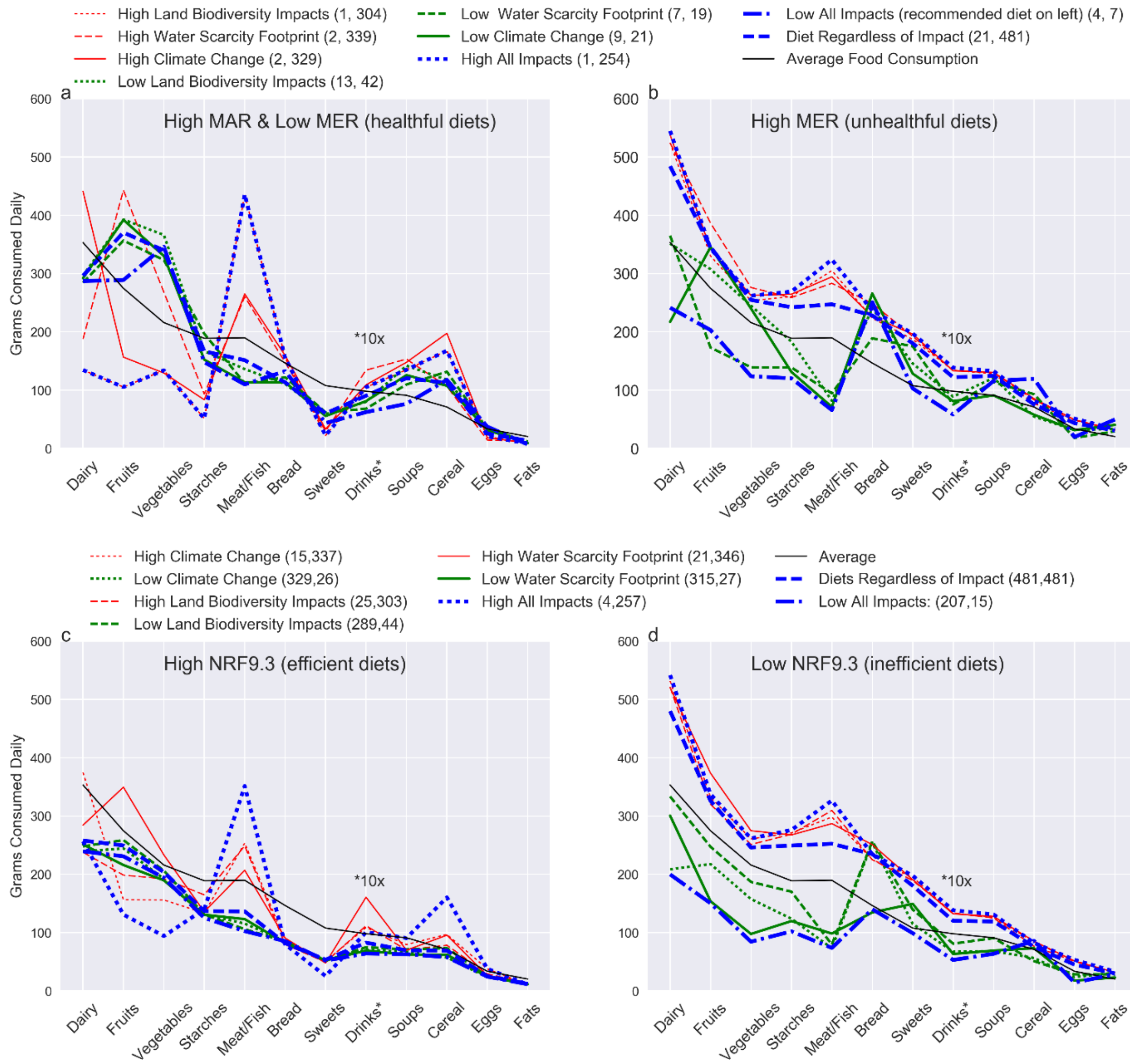

Figure S14a-d. Comparison of eating patterns between individuals, regardless of gender or country, falling in the top and bottom third for all impacts (≥ 6.5 and ≤ 4.8 kgCO2eq, ≥ 286 and ≤ 217 lit eq, and ≥ 5.14E-14 and ≤ 3.57E-14 PDF*yr, respectively), MAR shown in a-b (≥ 0.99 and ≤ 0.95, respectively), MER shown in a-b (≥ 1.6 and ≤ 1.2, respectively) and c-d shows NRF9.3 (≥ 0.23 and ≤ 0.15, respectively) for each of the food groups. Numbers in parentheses indicate the number of participants (out of 1457) that fall in this category for the left or right graph, respectively. The drinks category includes the water content of the evaluated beverages. *To improve visualization of the graphs, the grams of drinks consumed was divided by 10.

Table S3. Recommended percent changes in typical subset eating patterns to achieve a diet that is both high quality (as quantified by high MAR, low MER, and high NRF9.3) and low impacts (as quantified by low climate change, low biodiversity loss, and low water scarcity footprint). Green shading represents that increases in the food group consumption are required, red shading represents that decreases in the food group consumption are required.

	Bread and Savory Biscuits	Cereal	Drinks	Dairy	Eggs	Fats and Spreads	Fruits	Meat and Fish	Potatoes, Rice, and Pasta	Soups, Sauces, and Spreads	Sweets	Vegetables
German Male	-15.9	117.5	-44.6	-6.4	49.2	-71.8	5.8	-36.0	-31.8	6.6	-55.2	90.0
German Female	7.0	203.8	-40.9	-17.3	57.4	-67.5	-4.5	-9.7	-17.6	-1.8	-57.7	65.6
Greek Male	10.6	71.8	-10.2	2.0	13.6	-63.6	34.1	-45.1	-22.0	62.8	-52.0	80.7
Greek Female	23.4	132.3	-2.6	14.8	66.0	-66.1	20.8	-31.5	-1.7	58.3	-51.9	61.4
Irish Male	-18.6	12.4	-44.4	-11.6	-31.3	-74.1	10.5	-51.2	-36.2	-22.4	-69.1	75.2
Irish Female	29.5	-3.4	-30.8	-17.2	1.0	-62.9	-15.8	-39.1	-22.2	-31.8	-66.7	29.4
Dutch Male	-47.4	31.0	-52.8	-31.4	-2.4	-70.9	-1.2	-44.1	-31.2	-21.2	-55.7	66.4
Dutch Female	-32.4	100.4	-49.3	-17.0	81.4	-65.5	-1.0	-18.8	-9.2	-25.8	-50.2	69.6
Polish Male	-40.5	135.6	-38.5	-42.0	-13.4	-75.8	45.8	-55.4	-33.1	-40.6	-65.2	107.4
Polish Female	-13.1	104.5	-32.2	-16.4	26.7	-64.1	19.3	-29.7	-11.3	-34.8	-59.7	77.5
Spanish Male	-19.7	208.5	-32.0	-29.8	-5.5	-57.6	11.7	-66.0	-28.8	-12.3	-63.4	49.0
Spanish Female	11.1	157.9	-15.7	-30.8	7.7	-66.3	-10.1	-52.9	4.0	-7.7	-54.7	61.0
UK Male	16.4	7.4	-44.5	-24.2	-30.9	-55.8	-5.0	-48.0	-40.5	-33.2	-66.9	30.8
UK Female	32.1	48.4	-41.0	-18.8	6.4	-56.2	7.8	-33.2	-20.8	-19.3	-59.8	38.9
Vegetarian	5.0	28.4	-30.0	-6.9	-0.1	-55.8	-16.3		-13.5	-3.0	-51.6	-0.3
No Red Meat	12.5	2.2	-38.9	-15.8	-4.6	-63.5	-20.9	50.4	-12.4	-15.6	-51.2	8.1
No Dairy	-6.9	42.7	15.9	183.2	-75.0	16.0	-32.9	-45.3	-30.3	-55.5	-31.3	10.9
Male	-23.4	55.5	-40.7	-22.3	-6.2	-68.5	11.1	-51.4	-31.8	-14.6	-61.4	67.7
Female	3.7	72.6	-33.2	-16.0	27.3	-64.2	1.4	-33.1	-12.6	-16.8	-58.2	55.3
Total Average	-9.5	65.4	-36.5	-18.7	10.9	-66.1	5.2	-42.1	-21.7	-15.9	-59.6	60.2

S4. Supplementary Comparison of energy intakes:

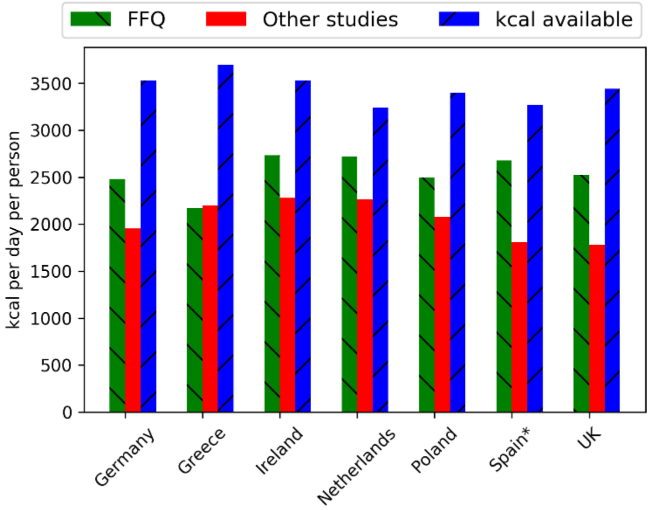

Figure S15. Daily energy intake (as kcal) using other nutrition studies and available kcal statistics. Other nutrition study's data available from (1)(2)(3)(4)(5)*(6), from left to right. Available kcal taken from FAO kcal availability per country (7). *indicates that under-reporters in this study were not removed, thereby lowering the average energy intake.

S5. Supplementary Scenario Analysis for Different Food Production Methods

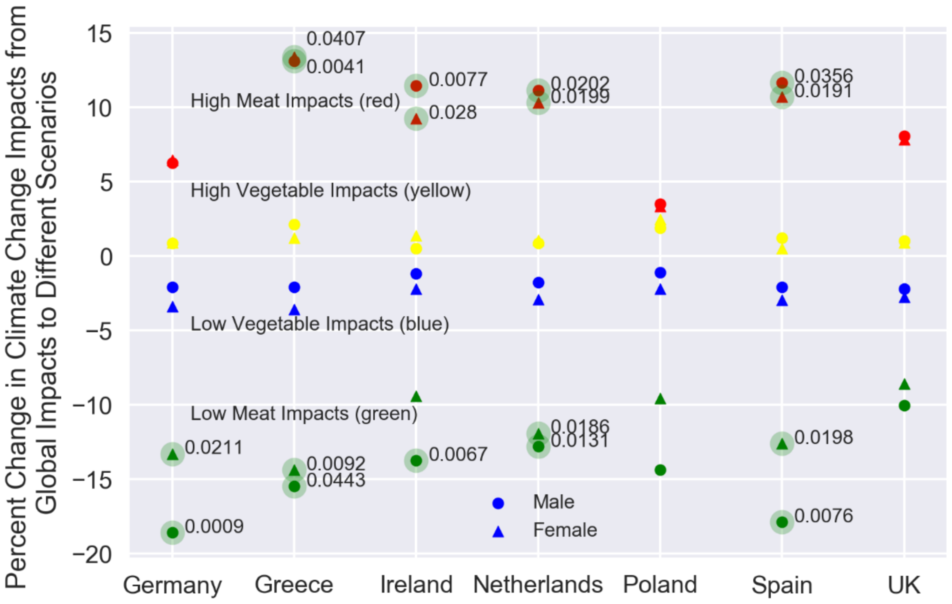

Figure S16. Percent changes in climate change impacts from global values when considering four different food choice scenarios. Significance from the new impact to the original, global impact is marked by a transparent green dot and p-values are printed next to the dot. Points with no transparent dot indicate that there was not a significant change in impacts from the global impact value scenario. Statistical significance between the different scenarios was verified using an unpaired two sided t-test under the assumption that p-values lower than 0.05 indicated statistically significant differences between the means of subsets.

S6. Supplementary Material for Data Sources, Nutrition, and Environmental Indicators

Food Consumption Data

Food consumption data was derived from the Food4Me study (8) taking place between 2012 and 2014. Food consumption was measured through the use of an online food frequency questionnaire (FFQ). The previous month's habitual food consumption was assessed by collected data on consumption frequency and portion size for various food and drink items (9)(10). The FFQ contained 162 food items (both single items and composite dishes), aggregated into 12 food groups, from which participants could choose. The study included over 1'400 men and women from seven European countries (Germany, Greece, Ireland, Netherlands, Spain, Poland, and the UK) between the ages of 18 and 79, with full details regarding age, gender, weight, health, physical activity levels, and reasons for participating in the study, published elsewhere (11). As the Food4Me study was intended to alter an individual's eating patterns based on personalized diet and nutrition advice, food consumption data from the baseline month, prior to recommendations for changing one's diet, was used.

Diet Quality Indicator

Daily nutrient intake values were based on the European Food Safety Authority's (EFSA) adequate intake (AI) dietary reference values (shown in Table S4) (12). AI values are based on experimental data and are the recommended average daily nutrient intake level to meet or exceed the needs of most healthy individuals (13). Because the population subset studied here is located in various countries throughout Europe, the AI values from EFSA (and not from an individual country) were used in calculating the nutrition indicators. However, gender and age specific RDA values published by the US National Institute of Medicine (13) were also considered. The sensitivities of the rate of consuming less than the recommended intake to the choice of dietary reference value (AI or RDA) is included in the supporting information (Table S5).

Table S4. Dietary Reference Values

		Recommended Daily Allowance (RDA) (13)		Adequate Intake (AI) (12)		Global Burden of Disease
NUTRIENT		MEN	WOMEN	MEN	WOMEN	Contribution to total DALYs
Beneficial Nutrients						
1	Protein (g/day)	56	46	56	47	0.04%**
2	Dietary Fiber (g/day)	38 (19-50)*<br>30 (51-70)*	25 (19-50)*<br>21 (51-70)*	25	25	0.56%**
3	Vitamin A (ug/day)	900	700	750	650	
4	Vitamin C (mg/day)	90	75	45	45	
5	Vitamin E (mg/day)	15	15	13	11	
6	Calcium (mg/day)	1000	1000 (19 –50)*<br>1200 (51-70)*	700	700	0.29%**
7	Iron (mg/day)	8	18 (19-50)*<br>8 (51-70)*	11	16	1.13%**
8	Magnesium (mg/day)	400 (19-30)*<br>420 (31-70)*	310 (19-30)*<br>320 (31-70)*	350	300	
9	Potassium (mg/day)	4700	4700	3100	3100	
10	Thiamin (mg/day)	1.2	1.1	1.1	0.9	
11	Riboflavin (mg/day)	1.3	1.1	1.6	1.3	
12	Niacin (mg/day)	16	14	18	14	
13	Vitamin B6 (mg/day)	1.3 (19 -50)*<br>1.7 (51-70)*	1.3 (19 -50)*<br>1.5 (51-70)*	1.7	1.6	
14	Folate (ug/day)	400	400	330	330	
15	Vit B12 (ug/day)	2.4	2.4	4	4	
16	Zinc (mg/day)	11	8	9.5	7	
17	Copper (mg/day)	0.9	0.9	1.1	1.1	
18	Iodine (ug/day)	150	150	130	130	0.09%**
19	Selenium (ug/day)	55	55	55	55	

Nutrients to Limit				
		Men	Women	
20	Saturated Fat (g/day)(14)<br>10% of total energy	26.7 (19-30)*<br>24.4 (31-50)*<br>22.2 (51-70)*	22.2 (19-30)*<br>20.0 (31-50)*<br>17.8 (51-70)*	1.58%***
21	Sugars (g/day) (14)<br>25% of total energy	150 (19-30)*<br>137.5 (31-50)*<br>125 (51-70)*	125 (19-30)*<br>112.5 (31-50)*<br>100 (51-70)*	
22	Sodium (mg/day) (15)	2300	2300	1.97%**
* values in parentheses indicate the age range for a given intake<br>** DALYs associated with dietary risk for under consumed protein, fiber, calcium, iron, and iodine or overconsumed sodium, respectively, for western Europe for 2015.<br>*** includes DALYs due to high intake of processed meats, trans fat, red meat, and sugar sweetened beverages.				

Table S5. Comparison of the number of people under-consuming a specific nutrient under the Adequate Intake value versus the Recommended Dietary Allowance

	Adequate Intake (European Food Safety Authority's (EFSA)		Recommended Dietary Allowance values published by the US National Institute of Medicine	
	Average MAR	Number of People under-consuming a nutrient (out of 1457)	Average MAR	Number of People under-consuming a nutrient (out of 1457)
Protein	1.45	312	2.11	54
Vitamin A	3.0	81	2.02	257
Thiamin	1.05	1096	2.27	111
Riboflavin	1.98	118	1.98	118
Niacin	2.09	77	1.77	157
Vitamin B6	1.92	85	1.95	90
Folate	1.48	312	0.92	977
Vitamin B12	2.01	226	3.36	50
Vitamin C	5.48	9	2.04	277
Vitamin E	0.98	900	0.77	1169
Calcium	2.27	70	1.2	593
Potassium	1.16	587	0.87	1043
Iron	1.20	671	1.52	553
Magnesium	1.2	547	1.1	714
Zinc	1.95	77	1.3	436
Copper	1.2	614	1.88	135
Iodine	1.86	211	1.24	585
Selenium	1.57	306	1.14	684
Dietary Fiber	1.18	641	1.07	806

The diet quality of an individual was measured using two absolute indicators and one efficiency indicator. The first absolute indicator, Mean Adequacy Ratio (MAR), has been developed as a measure of adequate nutrient consumption (16). This value correlates with nutrient deficiencies, and is calculated through the following equations:

$$NR_{en,i} = \frac{intake_{en,i}}{AI_{en,i}},\ NR_{en,i} = \{0 \dots 1\}$$

$$MAR = \frac{1}{19} * \sum_{i=1}^{19} NR_{en,i}$$

where the nutrient ratio (NR) is the ratio of the $intake_{en}$ (the daily consumed mass of a specific encouraged nutrient (en)) to the AI_{en}. Nutrients 1 through 19 in Table S4 were considered in this

calculation. The NR for each nutrient *i* was capped at one to avoid that overconsumption of one nutrient compensate for under supply of others in an individual's average MAR value. In Vieux's study (17), Vitamin D was also included as a nutrient in the calculation, however because it is also synthesized by the body upon skin exposure to sunlight, we have decided it should not be included in the calculation for a diet based indicator.

Because MAR does not capture consumption of nutrients that should be consumed in limited quantities, the Mean Excess Ratio (MER), as developed by (17) was also calculated for each individual using the equation below. Limiting nutrients (ln) considered in the MER calculation, as well as their maximum recommended values (MRV_{ln}), are shown as items 20 through 22 in Table S4. In the case of the MER calculation, NRs not reaching one were adjusted to one to avoid compensating for a higher intake of the other limiting nutrients.

$$NR_{ln,j} = \frac{intake_{ln,j}}{MRV_{ln,j}}, NR_{ln,j} = \{1 \dots inf\}$$

$$MER = \frac{1}{3} * \sum_{j=1}^{3} NR_{ln,j}$$

MRV limits for saturated fats were set to 10% of the total required daily energy consumption and for sugars were set to 25% of an individual's total required daily energy consumption (14). Sodium MRV was set to 2.3g per day (15)(14).

The Nutrient Rich Food Index 9.3 (NRF9.3) was used as an efficiency indicator to measure the nutritional quality of each diet and includes the combination of both beneficial and harmful nutrients as well as energy intake. It was found that NRF9.3 was a good indicator for identifying poor quality diets, as it correlated well with MER, but was not a good indicator to identify people who consumed less than recommended levels of beneficial nutrients. This was developed as a method of ranking the nutritional quality of foods and was found to be highly correlated to diet quality as measured through the Healthy Eating Index (HEI) (18). The nutrients included in the NRF9.3 were chosen by (18) because they showed the best correlation to the HEI when compared to other sets of nutrient combinations. For this indicator, the NR was set to a maximum of one for encouraged nutrients and set to a minimum of one for limiting nutrients, as in the MAR and MER calculations. Because the NRF value is not an average as the MAR and MER, it will change depending on the number of nutrients considered in the calculation and is relative to calorie intake. This indicator utilizes nine encouraged nutrients (Table S4 items 1 to 9) and three nutrients to limit (Table S4 items 20 through 22). The NRF9.3 was calculated using the following equation:

$$NRF9.3 = \frac{\sum_{i=1}^{9} NR_{en,i} - \sum_{j=1}^{3} NR_{ln,j}}{daily\ kcal\ intake}$$

Estimation of Diet-Related Environmental Impacts

Impact values per gram of food were calculated for each of the 162 foods/dishes on the FFQ. Composite foods were broken down into their three main ingredients by mass using a generic recipe or product label. Impacts were calculated for the mass of each ingredient and summed for a total impact per gram of each composite food. In many cases, impacts were available per crop type or ingredient (e.g. tomatoes) but not for a product (e.g. ketchup) derived from that crop. In this case, the impact associated with the root product (tomatoes) was determined and conversion factors, as provided in (19) were used to calculate the impact of the derived product. When impacts for derived products were available in databases or literature, these values were used in place of root products and conversion factors. A table showing the foods/dishes, their three main ingredients, conversion factors, associated processing energy and references (included only for climate change), and any assumptions is included in the Supplementary Electronic Table online.

The impact of each gram of food was calculated for climate change, WFP, and land-use driven biodiversity loss as follows: climate change impacts, measured as kg CO_2 equivalents (kgCO_2eq) per gram of food, were calculated using a combination of the Ecoinvent 3.3 database (20), the ZHAW database (21), and the AGRIBALYSE v1.2 database (www.ademe.fr) using IPCC GWP 2013 100 years characterization factors (22) with Brightway (23). The WFP, measured as liters equivalent (lit_{eq}) per gram of food, was calculated by multiplying a monthly, regional water stress index (24) with crop specific irrigation requirements to determine the global production-weighted water footprint per crop. Land-use driven biodiversity impacts were measured as global potentially disappeared fractions (PDF)*years per gram of food based on the crop specific, taxa aggregated impacts as defined in (25). In both the WFP and the biodiversity assessments, global weighted production averages were used, regardless of the country of consumption, to allow for an assessment of the impact due to varying diets and not to the changes in the supply chain.

WFP and land-use biodiversity loss impacts associated with livestock products (beef, chicken, milk, eggs, pig, sheep, and fish) were calculated based on the cultivation of animal feed required per gram of product using a combination of farming systems (global averages of extensive, intensive, or mixed production systems) for the specific livestock product. The fraction of concentrate feed (consisting of maize, wheat, barley, and soymeal) and the feed conversion efficiencies (using global values) were obtained from (26), with remaining feed assumed to be roughage and modeled as grass. The fraction of concentrate feed (consisting of maize, wheat, barley, and soymeal) and the feed conversion efficiencies (using global values) were obtained from (26), with remaining feed assumed to be forage with half modeled as harvested grass (25) and the other half modeled as pasture using the global characterization factor for pasture (27), the total available grassland (28), and a production rate of 1 kg/ha/yr. The ratios of the concentrate feed crops were modeled as specified per animal type as presented in (29). Biodiversity impacts due to fishing were not considered due to a lack of life cycle impact assessment methodology for aquatic biodiversity loss, therefore these impacts will be underestimated.

For each indicator, each individual's impacts were calculated by multiplying the impacts per gram of food/dish by the reported daily grams of the food consumed by that person. Details of the impacts for one gram of each food/dish type, the average daily grams consumed for each food/dish type, and the consumption weighted and unweighted average impacts for each of the food groups (eggs were excluded) are included in Figures S3 through S13. An environmental impact efficiency indicator, calculated as the ratio of impacts to energy intake, was also determined for each individual. This indicator shows the impacts associated with an individual's kcal consumption, regardless of the nutrients consumed, and can show whether primarily high impact or low impacts foods are consumed in relation to their energy intake.

REFERENCES:

1. Heuer T, Krems C, Moon K, Brombach C, Hoffmann I (2015) Food consumption of adults in Germany: results of the German National Nutrition Survey II based on diet history interviews. *Br J Nutr* 113(10):1603–1614.

2. Psaltopoulou T, et al. (2004) Olive oil, the Mediterranean diet, and arterial blood pressure: the Greek European Prospective Investigation into Cancer and Nutrition (EPIC) study. *Am J Clin Nutr* 80(0002–9165 (Print)):1012–1018.

3. Harrington J, et al. (2008) *SLÁN 2007: Survey of Lifestyle, Attitudes and Nutrition in Ireland. Dietary Habits of the Irish Population* (Dublin) Available at: http://epubs.rcsi.ie/cgi/viewcontent.cgi?article=1005&context=psycholrep.

4. TM van Rossum C, Fransen HP, Verkaik-Kloosterman J, JM Buurma-Rethans E, Ocké MC (2011) *Dutch National Food Consumption Survey, Diet of children and adults 7 to 69 years* Available at: http://www.rivm.nl/en/Topics/D/Dutch_National_Food_Consumption_Survey.

5. Elmadfa I, et al. (2009) *European Nutrition and Health Report 2009.* doi:10.1159/000242365.

6. Whitton C, et al. (2011) National Diet and Nutrition Survey: UK food consumption and nutrient intakes from the first year of the rolling programme and comparisons with previous surveys. *Br J Nutr* 106(12):1899–1914.

7. ChartsBin statistics collector team 2011 (2011) Daily Calorie Intake Per Capita. *ChartsBin.com*. Available at: http://chartsbin.com/view/1150 [Accessed October 17, 2016].

8. Celis-Morales C, et al. (2015) How reliable is internet-based self-reported identity, socio-demographic and obesity measures in European adults? *Genes Nutr* 10(5):28.

9. Forster H, Fallaize R, Gallagher C, et.al. (2014) Online dietary intake estimation: the Food4Me food frequency questionnaire. *J Med Internet Res* 16(6):e150.

10. Fallaize R, et al. (2014) Online Dietary Intake Estimation: Reproducibility and Validity of the Food4Me Food Frequency Questionnaire Against a 4-Day Weighed Food Record. *J Med Internet Res* 16(8):1–20.

11. Livingstone KM, Celis-Morales C, Navas-Carretero S, et.al. (2016) Profile of European adults interested in internet-based personalised nutrition: the Food4Me study. *Eur J Nutr* 55(2):759–769.

12. European Food Safety Authority (2010) European Food Safety Authority Dietary Reference Values and Dietary Guidelines. Available at: https://www.efsa.europa.eu/en/topics/topic/dietary-reference-values-and-dietary-guidelines [Accessed November 1, 2016].

13. Otten JJ, Hellwig JP, Linda D (2006) *DRI, dietary reference intakes: the essential guide to nutrient requirements* (National Academies Press, Washington, D.C.).

14. U.S. Department of Health and Human Services and U.S. Department of Agriculture (2016) 2015 – 2020 Dietary Guidelines for Americans. *8th Ed.*

15. Havas S, Roccella EJ, Lenfant C (2004) Reducing the Public Health Burden from Elevated Blood Pressure Levels in the United States by Lowering Intake of Dietary Sodium. *Am J Public Health* 94(1):19–22.

16. Tabacchi G, et al. (2009) How is the adequacy of micronutrient intake assessed across Europe? A systematic literature review. *Br J Nutr* 101(S2):S29-36.

17. Vieux F, Soler L-G, Touazi D, Darmon N (2013) High nutritional quality is not associated with low greenhouse gas emissions in self-selected diets of French adults. *Am J Clin Nutr* 97(9):569–583.

18. Fulgoni VL, Keast DR, Drewnowski A (2009) Development and validation of the nutrient-rich foods index: a tool to measure nutritional quality of foods. *J Nutr* 139(8):1549–1554.

19. Scherer L, Pfister S (2016) Global Biodiversity Loss by Freshwater Consumption and Eutrophication from Swiss Food Consumption. *Environ Sci Technol* 50(13):7019–7028.

20. Wernet G, et al. (2016) The ecoinvent database version 3 (part I): overview and methodology. *Int J Life Cycle Assess* 21(9):1218–1230.

21. Eymann L, Kreuzer S, Stucki M, Scharfy D (2014) Ökobilanz von Milch und Milchprodukten. *ZHAW Zürcher Hochschule für Angew Wissenschaften, IUNR Inst für Umwelt und Natürliche Ressourcen, Grüental, Postfach CH-8820 Wädenswil*.

22. IPCC (2013) Climate Change 2013: The Physical Science Basis. Contribution of Working Group I to the Fifth Assessment Report of the Intergovernmental Panel on Climate Change. *Intergov Panel Clim Chang Work Gr I Contrib to IPCC Fifth Assess Rep (AR5)(Cambridge Univ Press New York)*:1535.

23. Mutel CL (2017) *Brightway: An open source framework for Life Cycle Assessment* Available

at: https://doi.org/10.21105%2Fjoss.00236.

24. Pfister S, Bayer P (2014) Monthly water stress: spatially and temporally explicit consumptive water footprint of global crop production. *J Clean Prod* 73:52–62.

25. Chaudhary A, Pfister S, Hellweg S (2016) Spatially Explicit Analysis of Biodiversity Loss Due to Global Agriculture, Pasture and Forest Land Use from a Producer and Consumer Perspective. *Environ Sci Technol* 50(7):3928–3936.

26. Mekonnen MM, Hoekstra AY (2012) A Global Assessment of the Water Footprint of Farm Animal Products. *Ecosystems* 15(3):401–415.

27. United Nations Environment Programme (2016) *Global Guidance for Life Cycle Impact Assessment Indicators* Available at: http://www.lifecycleinitiative.org/training-resources/global-guidance-lcia-indicators-v-1/.

28. Food and Agriculture Organization of the United Nations (2017) *FAO Sustainable Grasslands Working Paper* Available at: http://www.fao.org/nr/sustainability/grassland/en/%0A [Accessed April 28, 2017].

29. Herrero M, et al. (2013) Biomass use, production, feed efficiencies, and greenhouse gas emissions from global livestock systems. *Proc Natl Acad Sci U S A* 110(52):20888–93.
